# Lipid-bilayer-assisted two-dimensional self-assembly of DNA origami nanostructures

**DOI:** 10.1038/ncomms9052

**Published:** 2015-08-27

**Authors:** Yuki Suzuki, Masayuki Endo, Hiroshi Sugiyama

**Affiliations:** 1 Department of Chemistry, Graduate School of Science, Kyoto University, Kitashirakawa-oiwakecho, Sakyo-ku, Kyoto 606-8502, Japan; 2 CREST, Japan Science and Technology Agency (JST), Sanbancho, Chiyoda-ku, Tokyo 102-0075, Japan; 3 Institute for Integrated Cell-Material Sciences (WPI-iCeMS), Kyoto University, Yoshida-ushinomiyacho, Sakyo-ku, Kyoto 606-8501, Japan

## Abstract

Self-assembly is a ubiquitous approach to the design and fabrication of novel supermolecular architectures. Here we report a strategy termed ‘lipid-bilayer-assisted self-assembly' that is used to assemble DNA origami nanostructures into two-dimensional lattices. DNA origami structures are electrostatically adsorbed onto a mica-supported zwitterionic lipid bilayer in the presence of divalent cations. We demonstrate that the bilayer-adsorbed origami units are mobile on the surface and self-assembled into large micrometre-sized lattices in their lateral dimensions. Using high-speed atomic force microscopy imaging, a variety of dynamic processes involved in the formation of the lattice, such as fusion, reorganization and defect filling, are successfully visualized. The surface modifiability of the assembled lattice is also demonstrated by *in situ* decoration with streptavidin molecules. Our approach provides a new strategy for preparing versatile scaffolds for nanofabrication and paves the way for organizing functional nanodevices in a micrometer space.

## Abstract (web summary)

Self-assembly is a useful method to fabricate novel supramolecular architectures. Here, the authors use lipid-bilayer-assisted self-assembly to obtain two-dimensional crystalline DNA origami lattices, imaging dynamic assembly phenomena using high-speed atomic force microscopy.

## Graphical abstract

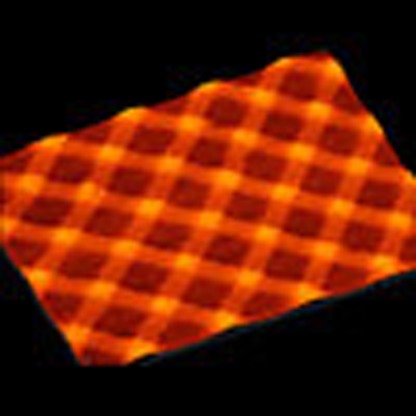

Self-assembly of nanostructural units into higher-order structures is now an indispensable approach for the bottom-up construction of nanoscale to microscale architectures^1^,^2^,^3^. To reveal the mechanisms that govern self-assembly, it is essential to understand the interplay between the properties of assembly units and the dynamics of the assembling process. Structural DNA nanotechnology, which is based on the inherent programmability of DNA helices, can provide versatile model units for self-assembly processes, as represented by the successful formation of various two-dimensional (2D)^4^,^5^ and three-dimensional structures^6^,^7^,^8^. The invention of the scaffolded DNA origami method^9^ further expanded the designability of DNA nanostructures^10^,^11^,^12^,^13^. Because of their high production yield, shape adoptability and surface or end modifiability, DNA origami nanostructures have been considered as ideal structural units for large-scale assemblies. Importantly, interactions between origamis can also be designed. In recent years, the creation of scaled-up origami assemblies was achieved via various approaches^14^,^15^,^16^,^17^,^18^,^19^,^20^,^21^,^22^,^23^,^24^, including the use of sticky-strand hybridization^15^,^16^,^17^,^18^,^19^,^22^ or blunt-end stacking^20^. However, despite this progress, the assembly of origami units into periodic lattices with micrometre-order dimensions remains a difficult challenge.

The major approach used towards the generation of larger DNA origami lattices has been the hierarchical assembly of preformed origami units in bulk solution^15^,^16^,^17^,^18^,^19^,^20^,^21^,^22^. However, as the second step of these approaches relies on specific hybridizations between origami units, the optimization of annealing conditions or strict temperature control is required to obtain the assemblies at high yield. In addition, the deposition of the solution-assembled large lattices onto a substrate while preventing the breakage and distortion of their structures is also challenging. In fact, there have been few successful examples of the production of 2D lattices with micrometre-order dimensions via hierarchical assembly in a test tube^18^. The alternative approach is surface-diffusion-mediated self-assembly at the liquid–solid interface^25^,^26^,^27^. Success in this approach requires a weak adsorption condition that allows molecular mobility on the surface and/or a dynamic adsorption–desorption equilibrium^28^. Rafat *et al.*^26^ recently demonstrated a mica-surface-assisted assembly of DNA origami structures by controlling the surface mobility of the origami units via the addition of Na^+^ ions. Woo *et al.*^27^ also succeeded in producing DNA origami chequerboard lattices via the stepwise control of the ionic strength of the buffer on the mica surface. However, these techniques require a high Na^+^ concentration, which is far from the conventional conditions that are used for folding and stabilizing DNA origami structures.

To achieve surface-assisted assembly, the selection of substrates is also crucial, in addition to buffer conditions. A synthetic zwitterionic lipid 1,2-dioleoyl-*sn*-glycero-3-phosphocholine (DOPC) is often used alone or in combination with other lipids to produce a planar mica-supported lipid bilayer (SLB), which is a popular model of biological membranes^29^,^30^. Because of their surface flatness, SLBs can be good candidates as substrates both for surface-assisted assembly^31^,^32^,^33^ and for atomic force microscopy (AFM) imaging^34^,^35^. It should also be noted that negatively charged unmodified DNA origami nanostructures can be electrostatically adsorbed onto a zwitterionic bilayer in the presence of divalent cations^36^,^37^,^38^, such as Mg^2+^ and Ca^2+^.

On the basis of these features, we report herein lipid-bilayer-assisted 2D self-assembly of DNA origami structures. A mica-supported zwitterionic DOPC lipid bilayer is used as a substrate to allow electrostatic adhesion and self-organization/reorganization of DNA origami structures in the conventional buffer solution optimized for the preparation of origami structures. We demonstrate that prefolded origami units can be readily deposited onto the SLB and assemble into ordered lattices on it even in the absence of any buffer exchange processes.

## Results

### Blunt-end-stacking-mediated lattice formation

**Figure 1:**
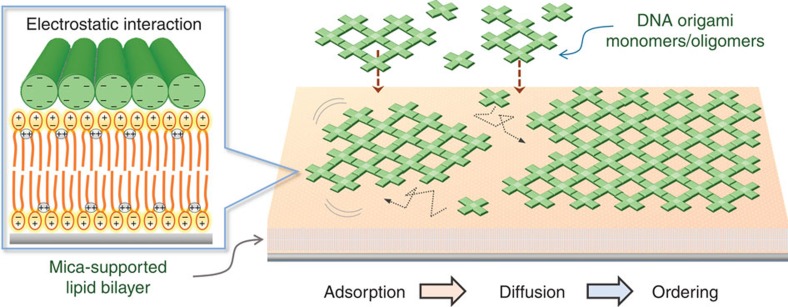
Schematic representation of the lipid-bilayer-assisted self-assembly of DNA origami nanostructures. Interaction between a DNA origami and a mica-supported lipid bilayer is mediated by divalent cations. Surface-mobile DNA origami structures can self-assemble into lattices.

Our strategy for the lipid-bilayer-assisted self-assembly is schematically illustrated in Fig. 1. DNA origami structures that are absorbed weakly onto a zwitterionic SLB by Mg^2+^-mediated interaction can retain mobility, and thus associate to form ordered superstructures on the surface.

**Figure 2:**
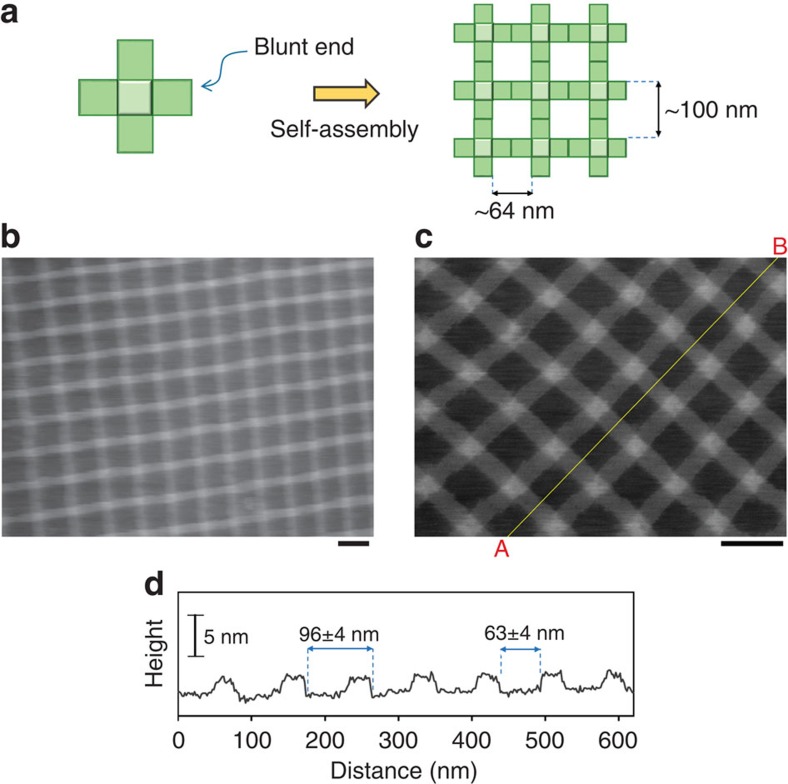
Lattice formation from cross-shaped DNA origami units. (**a**) Each edge of the cross-shaped DNA origami structure was designed to have a blunt end, thus enabling stacking interactions between the origamis. (**b**,**c**) AFM images of the lattice made from the cross-shaped DNA origamis. (**d**) Cross-sectional profile along the A–B line in (**c**). Scale bars, 100 nm.

Substrate-supported self-assembly is commonly achieved via relatively weak interactions^28^, such as van der Waals interactions, hydrogen bonds^39^,^40^,^41^ and π stacking^42^. As an assembly unit for 2D lattices, we first employed a twist-corrected cross-shaped DNA origami with blunt ends (Fig. 2a). The connection between the origami units was mediated by blunt-end stacking interactions, which enables the reorganization of growing crystals on the SLB by the reversible association and dissociation of origami structures^26^,^27^(Figs 1 and 2a).

The cross-shaped origamis were deposited onto an SLB that was prepared from DOPC (Methods; see also Supplementary Fig. 1) and incubated for 60 min in a buffer containing 20 mM Tris-HCl (pH 7.6), 10 mM MgCl_2_ and 1 mM EDTA (standard buffer). Note that this is the same buffer that was used for the preparation of our DNA origami structures. Figure 2b,c show the resulting ordered array of the cross-shaped units (see also Supplementary Fig. 2). The cross-sectional profile along the A–B line in Fig. 2c is shown in Fig. 2d, and demonstrates the periodic feature of the crystal. The periodicity along this line was 96±4 (mean±s.d.) nm, which agreed well with the expected value (Fig. 2a). The average height of the single-layered domains of the structural unit above the bilayer surface was about 2 nm, which was consistent with the theoretical value for the 2D DNA origami structure, indicating the formation of a monolayer (2D) lattice.

The assembled lattice was easily detached from the bilayer surface by the addition of NaCl (Supplementary Fig. 3a). After the addition of NaCl (final concentration, 200 mM), the lattice structures disappeared immediately from the scanning area. Importantly, this effect was reversible; that is, the bilayer surface allowed the adsorption of DNA origamis and the formation of lattices after changing the buffer again to standard one (Supplementary Fig. 3b,c). The same effect on adsorption/desorption was also observed when ionic strength was changed by the addition of KCl (Supplementary Fig. 3d). These results indicate that the interaction between origami units and DOPC bilayers is electrostatic and can be weakened by the addition of NaCl or KCl.

### Direct observation of the self-assembly process

To follow the formation of the lattice, next we performed dynamic real-space visualization using high-speed atomic force microscopy (HS-AFM). Cross-shaped origamis were often observed as oligomers (Supplementary Fig. 4a), even when images were obtained immediately after the deposition onto the bilayer surfaces (Supplementary Fig. 4b). This indicates that cross-shaped origamis can assemble to form oligomers or small lattices by stacking interactions in solution, as reported previously^26^; therefore, the formation of large lattices should involve the connection of these random-shaped structures.

**Figure 3:**
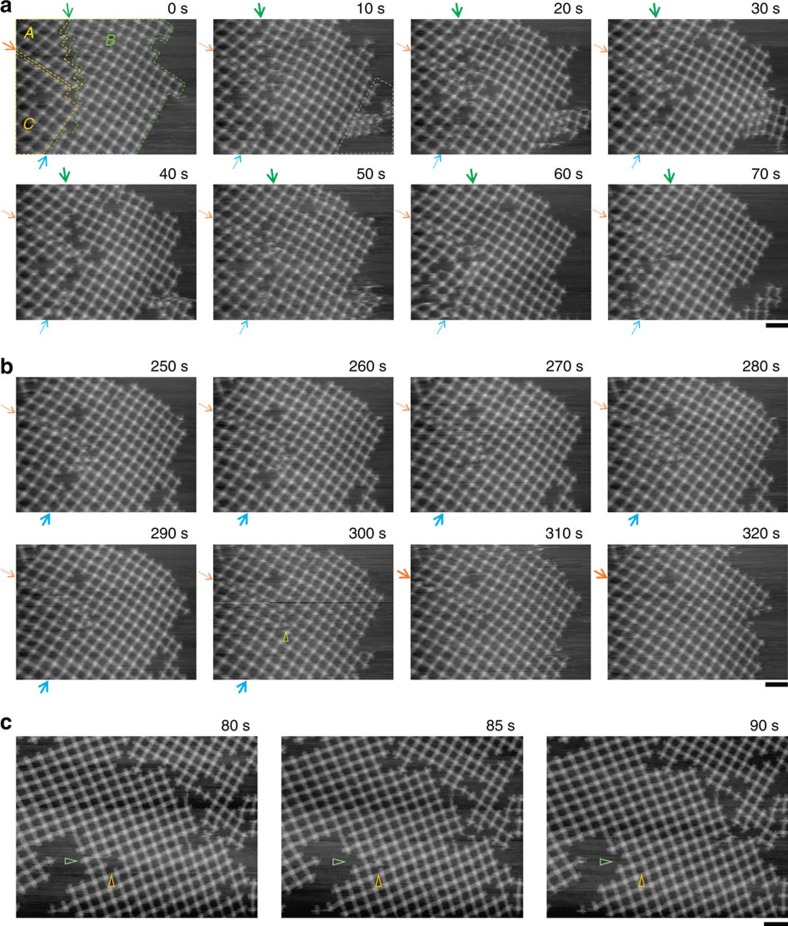
Processes of lattice formation. Time-lapse images of growing lattices on the SLB surface were obtained at 0.2 frames per s. (**a**,**b**) Lattice fusions. The elapsed time is shown in each image. In the initial frame of the consecutive images, three lattices named *A* (red framed), *B* (blue framed) and *C* (yellow framed) were observed in the scanning area. (**a**) Fusion of lattices *A* and *B*. (**b**) Fusion of lattices *B* and *C*. Gaps between lattices *A* and *B*, *B* and *C*, and *C* and *A* are indicated by green, blue and orange arrows, respectively. Association-and-dissociation-coupled reorganization was also observed at the right boundary of lattice *B* (dashed purple area). Excess units between the *B* and *C* boundaries were desorbed away to allow a complete connection (yellow triangle). Details are seen in Supplementary Movie 1. (**c**) Defect filling. The elapsed time is shown in each image. The point defect (orange triangle) was filled with the monomer came from the lattice edge (green triangle). Details are seen in Supplementary Movie 2. Scale bars, 200 nm.

Successive HS-AFM images revealed a variety of fundamental processes of the lattice growth. Figure 3 shows representative time-lapse AFM images. In the initial frame, there were three lattices in the scanning area of 1,600 × 1,200 nm^2^. Here, in Fig. 3a, we named them lattice ‘*A* (yellow-framed region in Fig. 3a)', ‘*B* (green-framed region in Fig. 3a)' and ‘*C* (orange-framed region in Fig. 3a)' for convenience. These three lattices became a large single lattice via a reorganization-coupled connection (Fig. 3a,b; see also Supplementary Movie 1). During the process of the connection between the *A* and *B* lattices (Fig. 3a, 0–70 s, green arrows) and the subsequent connection between the *B* and *C* lattices (Fig. 3b, 250–320 s, blue arrows), the association and dissociation of monomers and/or small oligomers occurred repeatedly at gaps between lattices, to produce ‘matched' boundaries. Interestingly, we observed that excess units between the boundaries were kicked out to allow the complete connection (Fig. 3b, 300–310 s, yellow triangle). From the initial frame of the sequential images, boundaries that matched each other were observed at the gap between *A* and *C*; however, they were engaged with each other to form a zipper-like pattern (Fig. 3a). This distortion was also solved at almost the same time as the completion of the *B*–*C* connection (Fig. 3b, 310–320 s, orange arrows). Interestingly, throughout the consecutive images, dynamic morphological changes caused by the association and dissociation of small oligomers were also observed at the right boundary of lattice *B* (Fig. 3a, 10 s, dashed purple area). These direct observations demonstrated the involvement of dynamic trial-and-error interactions in the growing process and support their occurrence at the bilayer surface.

The point defect at 320 s in Fig. 3b remained unfilled during the observation period. However, in the other example of the consecutive observation, filling of a point defect was successfully monitored (Fig. 3c, see also Supplementary Movie 2). The larger lattice in Fig. 3c has a point defect inside (Fig. 3c. 80 s, orange triangle). Similarly to the above example, association-and-dissociation-coupled reorganizations were observed especially at around boundary of the lattice. Intriguingly, in this observation one of the monomers dissociated from the lattice edge (Fig. 3c, green triangle) was seen to jump into the defect (Fig. 3c, 85 s) to fill it (Fig. 3c, 90 s). This indicates that defects arose in the growing process can be filled with free monomers.

Through the above observed phenomena, such as boundary-reorganization-coupled fusions and defect filling, the 2D DNA origami assembly rearrange at the bilayer surface to form a perfect ordered network. The same was not observed when the same origami solution was deposited onto the solid mica surface, where small patches remained disordered (Supplementary Fig. 1d,e). This difference can be related to the lateral diffusion of DNA origami on the SLB. It should be noted that solution-phase incubation of this cross-shaped origami often results in aggregation (Supplementary Fig. 5) due to spontaneous inter-/intramolecular stacking interactions in three-dimensional space. The SLB surface may contribute a stabilization against such aggregation, which also enables the extension of networks at the interface between the bilayer and solution.

### Surface modifiability of the SLB-supported lattice

**Figure 4:**
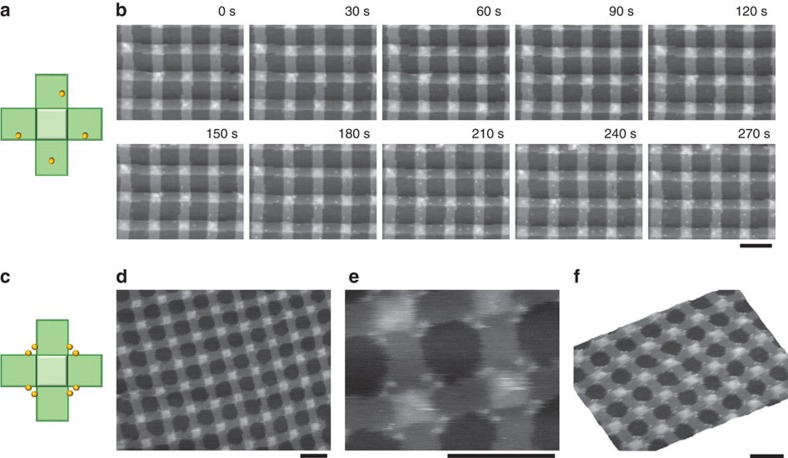
Surface modification of the lattice. (**a**) Design of a cross-shaped DNA origami structure carrying four biotinylated staple strands (yellow dots). The top face of the origami has four biotin moieties. (**b**) Time-lapse AFM images of the modification of the lattice with streptavidin molecules. While scanning of the same area was ongoing, 15 μl of the folding buffer containing 20 μM streptavidin was injected into 135 μl of the standard buffer (20 mM Tris buffer (pH 7.6), 1 mM EDTA and 10 mM MgCl_2_), so that the final concentration of streptavidin was 2 μM. Images were obtained at a scan rate of 0.2 frames per s. The elapsed time is shown in each image. The solution of streptavidin was added at 35 s. Details are seen in Supplementary Movie 3. (**c**) Design of a cross-shaped DNA origami structure carrying eight biotinylated staple strands (yellow dots). (**d**,**e**) AFM images of the lattice after modification with streptavidin (2 μM). (**f**) A topographic AFM image of the streptavidin-modified DNA origami lattice. Scale bars, 100 nm.

To investigate the surface modifiability of the bilayer-supported origami lattice, we prepared a derivative of the cross-shaped origami unit in which each arm had a single biotinylated staple (Fig. 4a). Four biotin moieties were designed to point to the same side of the origami, and their asymmetric positioning enabled us to identify the orientation of origami units in the lattice. The formation of the lattice of functionalized origami units on the SLB was first confirmed by AFM imaging; subsequently, a streptavidin solution was injected into the imaging buffer while scanning of the same area was ongoing. Figure 4b shows the representative image set of this *in situ* modification (see also Supplementary Movie 3). After 65 s of exposure of the streptavidin solution, binding events were observed. In this example, four origami units in the scanning area were fully decorated with streptavidin molecules (Supplementary Fig. 6a). Noteworthy, all of them were recognized as having a ‘facing up' orientation (that is, with the biotinylated side in the upward orientation). AFM images of the other areas after the modification, and their statistical analysis revealed that 47% of the origami units had four streptavidin molecules. Among them, 95% were recognized as having a ‘facing up' orientation (Supplementary Fig. 6b). Note that a high occupancy (92%) of binding sites was achieved when biotinylated origami was incubated with streptavidin in a test tube (Supplementary Fig. 7). Considering this fact, the unbound units reflected a ‘facing down' orientation rather than the misincorporation of biotinylated staples. The appearance of modified units with one to three streptavidin molecules was probably due to the capability of the biotinylated staples to thread to the other side of the origami sheet^43^. Therefore, we concluded that the orientation of the two sides on the SLB cannot be controlled in our method and appeared almost equal probabilities; however, the bilayer-supported lattice could be modified *in situ* as long as binding sites were accessible.

Encouraged by these results, next we aimed to produce a symmetric pattern of streptavidin along the lattices via the symmetric labelling of the origami units with biotin. For this purpose, lattices were prepared from another version of the biotinylated origami. In this version, each corner of the centre square of the cross was designed to have two biotin moieties surrounding it, thus allowing symmetric decoration with streptavidin molecules (Fig. 4c). The assembled lattices were directly modified *in situ* in the same manner as that described above (Supplementary Fig. 8a; see also Supplementary Movie 4). As shown in Fig. 4d–f, modification of the lattice created using this design resulted in a highly symmetric arrangement of streptavidin molecules. Specific binding to the biotinylated position was achieved at a yield of 94% (Supplementary Fig. 8b,c). It is also noteworthy that nonspecific binding of streptavidin molecules to the bilayer surface was not observed. The high yield and high specificity of the modification would be applicable to the alignment of the various streptavidin-conjugated or biotinylated molecules on the lattice surface.

### Close packing of symmetric DNA origami structures

**Figure 5:**
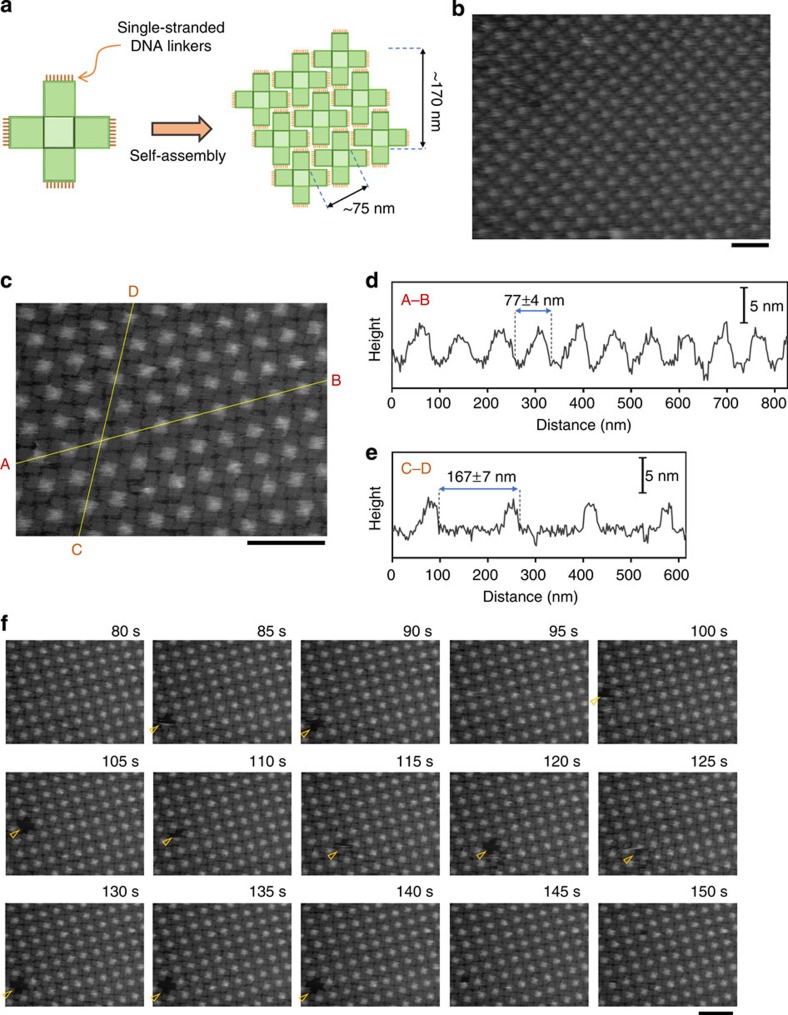
Close packing of cross-shaped DNA origami nanostructures. (**a**) Design of the cross-shaped DNA origami structure that was used for close packing. Stacking interactions between origamis were prevented by adding poly-T (four consecutive thymine bases) single-stranded DNA tails to all four edges. (**b**,**c**) AFM images of the close-packed cross-shaped DNA origami nanostructures. (**d**,**e**) Cross-sectional profiles along lines A–B (**d**) and C–D (**e**) in (**c**). (**f**) Successive HS-AFM images of close-packed cross-shaped origami and the defects therein. Images were obtained at a scan rate of 0.2 frames per s. The elapsed time is shown in each image. Details are seen in Supplementary Movie 5. Scale bars, 200 nm.

Next, we tested whether our method was applicable to the production of close-packed cross-shaped DNA origami structures. To prevent stacking-mediated interactions, each of the four edges of the cross-shaped origami was modified to have 12 T4 (four consecutive thymine bases) single-stranded DNA tails (Fig. 5a). As shown in Fig. 5b, incubation for 15 min on the SLB resulted in close-packed origami lattices, the pattern of which was different from that of the stacking-mediated assemblies presented in Fig. 1. The arms of a cross-shaped unit were engaged with the arms of adjacent units, to tessellate the SLB surface. The cross-sectional profiles along the A–B and C–D lines shown in Fig. 5c revealed that the periodicity along these lines was 77±4 and 167±7 nm, respectively (Fig. 5d,e). These values matched the expected values of 75 and 170 nm, respectively. In our experimental condition, this type of close-packed structure was only observed on SLB surfaces (Supplementary Figs 9 and 10). As shown in Fig. 5c, the surface was densely covered with cross-shaped origamis; however, we found defects in the lattice in some images, such as that depicted in Fig. 5b (see also Supplementary Fig. 11a).

Interestingly, we successfully monitored the defect formation and its healing within the close-packed structures using HS-AFM imaging. Figure 5f shows images of the close-packed origami lattice and the appearance of defects therein. The defect that appeared at 85 s seemed to be filled up directly with a monomer from the bulk solution. Conversely, the defect that appeared at 105 s diffused in the lattice and finally filled it (Fig. 5f; see also Supplementary Movie 5). These visualizations demonstrated the manner in which the lattices are maintained at the liquid–bilayer interface.

**Figure 6:**
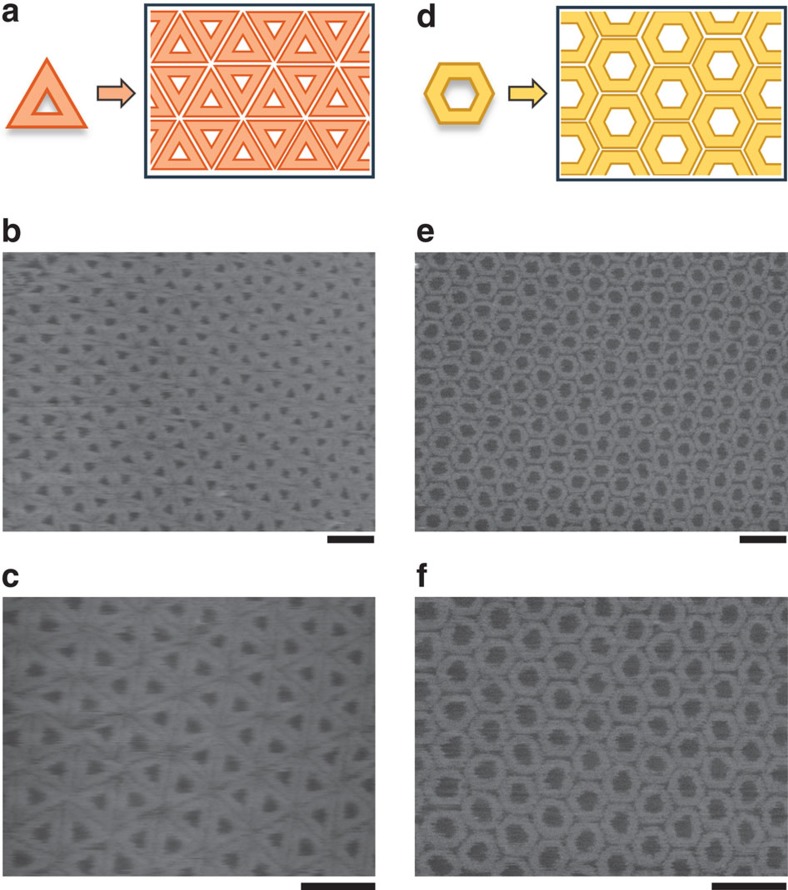
Close packing of symmetric DNA origami nanostructures. (**a**) A scheme of a close packing of triangular DNA origami nanostructures. (**b**,**c**) AFM images of the close-packed triangular DNA origami nanostructures. (**d**) A scheme of a close packing of hexagonal DNA origami nanostructures. (**e**,**f**) AFM images of the close-packed hexagonal DNA origami nanostructures. Scale bars, 200 nm.

Other types of symmetric origami structures, such as triangles^9^ or hexagons^22^, can also be ordered to close-packed lattices using the same procedure (Fig. 6 and Supplementary Fig. 11b,c). In Fig. 6b,c, the SLB surface was fully covered with hexagonally arranged triangular DNA origamis. Hexagonal DNA origami nanostructures were also close packed into a honeycomb pattern on the SLB surface (Fig. 6d–f). Fast Fourier transformation analyses of the images clearly demonstrated the symmetry of these lattices (Supplementary Fig. 12). In both cases, highly ordered arrays were observed over a range of 1.2 × 1.2 μm^2^.

Concentration of origami solution is obviously an important factor to obtain close-packed structures (Supplementary Fig. 13; see also Supplementary Movies 6 and 7). It was appeared that origami units moves randomly when amount of origamis were not enough to tessellate the SLB surfaces. The increase of [Na^+^] or decrease of [Mg^2+^] severely affected the close packing (Supplementary Figs 14–16). As [Na^+^] increased or [Mg^2+^] decreased, the packing became looser: origami units desorbed from the SLB surface more frequently, leading to an increase in the diffusibility of the units that remained on the surface (Supplementary Figs 14 and 16). An increase in [Na^+^] to 100 mM resulted in complete desorption of origami structures from the SLB surface (Supplementary Fig. 14g–l), as in the case of stacking-mediated lattices. Note that this condition still permitted origamis to remain on a bare mica surface. Further increase in [Na^+^] was required to detach them from the surface (Supplementary Fig. 15). On the other hand, close-packed structures were observed even after an increase in [MgCl_2_] to 20 mM (Supplementary Fig. 17). However, under this condition, two-layered origami structures were often formed in the lattices probably due to Mg^2+^-mediated attractive interaction between bulk-remained origamis and bilayer-supported origami lattices. These results indicate that electrostatic interaction has to be balanced from the following points: (i) enough amounts of origamis must be adsorbed to the SLB surface, but they should retain mobility on the surface for shape-matching-based self-organization/reorganization; (ii) at the same time, interaction between the origami units have to be strong enough to stabilize their adjacent positioning but weak enough to prevent layer-to-layer interaction of origamis.

## Discussion

We demonstrated the 2D self-assembly of DNA origami nanostructures on a lipid bilayer surface. In typical AFM imaging experiments, DNA origami nanostructures are strongly adsorbed and immobilized on negatively charged mica surfaces with the aid of buffer-derived Mg^2+^ ions. Thus, for the purpose of mica-supported self-assembly, hundreds of mM of Na^+^ have been used to induce the surface mobility of origamis^26^,^27^. We paid attention to the fact that the strength of the binding of DNA origamis to the DOPC bilayer surface was significantly weaker than that observed for the mica surface in the buffer condition optimized for the preparation of DNA origami (Supplementary Figs 9 and 15). This condition allowed the lateral diffusion of DNA origamis on the SLB surface, which resulted in 2D lattices via interactions between the origami units. The lattices can be formed either by blunt-end stacking interactions or by the close packing of symmetric DNA origami structures, including triangles and hexagons. The dynamic reorganization and healing (defect filling) involved in the lattice growth and/or maintenance were directly revealed by time-lapse AFM imaging.

It is important to note here that the physical properties of bilayers (that is, softness, elasticity and fluidity) are different from those of solid supports, such as mica. In the present study, we used solely the DOPC bilayer as a support for crystalline DNA origami lattices. However, SLBs are composition-tunable systems, in which various physicochemical properties of bilayers can be produced from different lipid species. Future studies on how the formation of 2D lattices depends on the properties of the bilayer will further enhance our understanding of the mechanisms that underlie the lipid-bilayer-assisted self-assembly.

We believe that our bilayer-supported origami lattices will serve as a versatile platform for a diverse range of applications. The lattices were directly modified *in situ*, which resulted in a large-scale array of streptavidin molecules. This surface accessibility should be applicable to the introduction of other components in the lattices. Considering that even dynamic systems, such as motors^44^,^45^ and cascade enzymatic reactions^46^, can be organized on individual DNA origami nanostructures, our assembly strategy may provide a route to producing arrays of these nanodevices and organizing them into more sophisticated circuits. Importantly, DNA origami structures placed on lipid bilayers can be manipulated by strand-exchange reactions^47^ or photo reactions^35^. A popular approach to anchor DNA nanostructures onto lipid membranes has been modification of strands with hydrophobic groups such as porphyrin^48^,^49^, glyceryl-bis-C_16_ (ref. 50), ethyl phosporothioate^51^ and cholesterol^52^,^53^. Employing these modifications, a variety of membrane-spanning^49^,^51^,^54^/binding^55^ or lipid-conjugated DNA nanostructures^56^ have been constructed in an attempt to mimic cellular membrane proteins or viral structures. In this context, our lattice structures on the lipid bilayer can be a mimic of membrane–cytoskeleton network structures, the function of which is implicated in the regulation of membrane protein function^57^. Using the nanosized cavities of our lattices, ordered arrangement of isolated membrane proteins may be achieved. It should also be emphasized that our method does not rely on the modification of staple strands with hydrophobic moieties; therefore, lattices are not anchored onto the bilayer. This feature will enable us to transfer the preassembled lattices onto the surface of other substrates in combination with lithographic methods^14^,^58^. We anticipate that our approach will further expand the potential applications of DNA origami structures and their assemblies in the fields of nanotechnology, biophysics and synthetic biology.

## Methods

### Materials

All staple DNAs used to prepare the DNA hexagonal unit were purchased from Operon Biotechnology (Tokyo, Japan). Single-stranded M13mp18 viral DNA was purchased from New England Biolabs, Inc (Ipswich, MA). The gel-filtration column and the Sephacryl S-300 were purchased from BioRad Laboratories (Hercules, CA) and GE Healthcare (Buckinghamshire, UK), respectively. Tris-HCl, EDTA and MgCl_2_ for electrophoresis analysis were purchased from Nacalai Tesque, Inc. (Kyoto, Japan). Water was deionized (18.0 MΩ cm specific resistance) by a Milli-Q system (Millipore Corp., Bedford, MA). Streptavidin was purchased from Wako Pure Chemical Industries (Osaka, Japan). DOPC was obtained from Avanti Polar Lipids (Alabaster, AL) as chloroform stocks.

### Preparation of DNA origami structures

The DNA origami structures were designed using the caDNAno software^59^,^60^ (Supplementary Figs 18–20 and Supplementary Tables 1–4). The origami unit was assembled in 20 μl of solution containing 10 nM M13mp18 single-stranded DNA, 40 nM staple DNAs (4 equiv.), 20 mM Tris buffer (pH 7.6), 1 mM EDTA and 10 mM MgCl_2_. For the preparation of the cross-shaped origami and its derivatives, the mixture was annealed by reducing the temperature from 85 to 65 °C at a rate of −1.0 °C min^−1^, and then from 65 to 15 °C at a rate of −0.5 °C min^−1^. For the preparation of the triangular and hexagonal origamis, the mixture was annealed by reducing the temperature from 85 to 15 °C at a rate of −1.0 °C min^−1^. Origami solutions were purified using a Sephacryl S-300 gel-filtration column after annealing was completed.

### Preparation of mica-supported lipid bilayers

SLBs were prepared from DOPC liposomes via the vesicle-fusion method^30^,^61^,^62^. Vesicles were prepared from a chloroform stock of DOPC. The chloroform was evaporated under a stream of nitrogen gas and the lipids were rehydrated overnight in water (from a Millipore water purification system), to give a total lipid concentration of 2 mg m^−1^ l^−1^. The lipid mixture was vortexed to produce large multilamellar vesicles, from which small unilamellar vesicles were prepared by sonication. Supported lipid bilayers were formed by depositing 2 μl of vesicle solution, followed by 1 μl of buffer (20 mM Tris buffer (pH 7.6), 1 mM EDTA and 10 mM MgCl_2_) onto freshly cleaved mica disks with a diameter of 1.5 mm (Furuuchi Chemical, Tokyo, Japan). To prevent the drying of the bilayers, the sample was incubated at room temperature in the sealed container, in the inside of which a piece of Kimwipe wetted with Milli-Q water is stuffed^63^. After 30 min of adsorption on mica, the sample was rinsed with the buffer to remove unadsorbed liposomes. The bilayers had a featureless flat surface, and sectioning through regions containing both the bilayer and bare mica indicated that the bilayer was ∼3.5 nm in thickness (Supplementary Fig. 1), as expected for a single bilayer^64^. Although the existence of bare mica regions is convenient for confirmation of the formation of SLB, an inhomogeneous surface is obviously unfavourable for the formation of a large lattice (Supplementary Figs 1 and 9). Therefore, the above mentioned procedure, from deposition to rinsing, was repeated twice to fill these gaps and cover the mica surface completely with a bilayer. Unless otherwise mentioned, SLBs prepared from DOPC alone were used in this study.

### Lattice formation on lipid bilayer surfaces

A drop (2 μl) of DNA origami nanostructures with designated shapes in the standard buffer (20 mM Tris buffer (pH 7.6), 1 mM EDTA and 10 mM MgCl_2_) solution (10 nM) was deposited onto the preformed SLB. The sample was incubated for 60 min (for stacking-mediated lattices) or 15 min (for close-packed structures) at room temperature in the sealed container described above. After the incubation, the surface was directly imaged by AFM in ∼150 μl of the standard buffer without surface rinsing. For the monitoring of streptavidin binding or NaCl-induced changes, imaging was started in 135 μl of the standard buffer, followed by injection of 15 μl of the buffer containing the designated concentration of streptavidin or NaCl.

### AFM imaging

AFM imaging was performed using a high-speed AFM system^65^,^66^ (Nano Live Vision, RIBM, Tsukuba, Japan) or a tip-scan type of high-speed AFM^67^,^68^ (BIXAM, Olympus, Tokyo, Japan) with a silicon nitride cantilever (resonant frequency=1.0–1.5 MHz in air, spring constant=0.1 N m^−1^, electron beam deposited (EBD) tip radius <10 nm; Olympus BL-AC10EGS-A2). Typically, 320 × 240 pixels images were obtained at a scan rate of 0.2 frames per second for time-lapse imaging. AFM images were analysed using the AFM Scanning System Software (Olympus, Tokyo, Japan) and ImageJ (http://rsbweb.nih.gov/ij/).

## Additional information

**How to cite this article:** Suzuki, Y. *et al.* Lipid-bilayer-assisted two-dimensional self-assembly of DNA origami nanostructures. *Nat. Commun.* 6:8052 doi: 10.1038/ncomms9052 (2015).

## Supplementary Material

Supplementary Figures and Tables Supplementary Figures 1-20 and Supplementary Tables 1-4

Supplementary Movie 1 Lattice formation on the SLB. Scanning rate: 0.2 frames per second, Image size: 1600 nm × 1200 nm. Movie is played ten times faster.

Supplementary Movie 2 Defect filling within the lattice. Scanning rate: 0.2 frames per second, Image size: 2000 nm × 1500 nm. Movie is played ten times faster.

Supplementary Movie 3 The lattice modification with streptavidin molecules. While scanning of the same area is continued, 15 μL of the standard buffer containing 20 μM streptavidin was injected to the 135 μL imaging buffer (20 mM Tris buffer (pH 7.6), 1 mM EDTA, and 10 mM MgCl2), so that final concentration of streptavidin was 2 μM. The injection was performed at 35 s. Scanning rate: 0.2 frames per second, Image size: 400 nm × 300 nm. Movie is played ten times faster.

Supplementary Movie 4 The lattice modification with streptavidin molecules. While scanning of the same area is continued, 15 μL of the standard buffer containing 20 μM streptavidin was injected to the 135 μL imaging buffer (20 mM Tris buffer (pH 7.6), 1 mM EDTA, and 10 mM MgCl2), so that final concentration of streptavidin was 2 μM. The injection was performed at 65 s. Scanning rate: 0.2 frames per second, Image size: 400 nm × 300 nm. Movie is played ten times faster.

Supplementary Movie 5 Defects formation and their filling within the close packed structure. Scanning rate: 0.2 frames per second, Image size: 800 nm × 600 nm. Movie is played ten times faster.

Supplementary Movie 6 Diffusion of cross-shaped DNA origamis with T4 tails on the SLB surface. The SLB surface was treated with 1 nM of the origami solution. Scanning rate: 1.0 frame per second, Image size: 800 nm × 600 nm. Movie is played two times faster.

Supplementary Movie 7 Diffusion of cross-shaped DNA origamis with T4 tails on the SLB surface. The SLB surface was treated with 5 nM of the origami solution. Scanning rate: 0.2 frames per second , Image size: 800 nm × 600 nm. Movie is played ten times faster.
