# Supplementary material for: Lipid-bilayer-assisted two-dimensional self-assembly of DNA origami nanostructures
Source: Nat Commun. 2015 Aug 27;6:8052. doi: 10.1038/ncomms9052 (PMC4560778; doi:10.1038/ncomms9052)
Supplement: Supplementary Figures and Tables — Supplementary Figures 1-20 and Supplementary Tables 1-4 [file ncomms9052-s1.pdf]

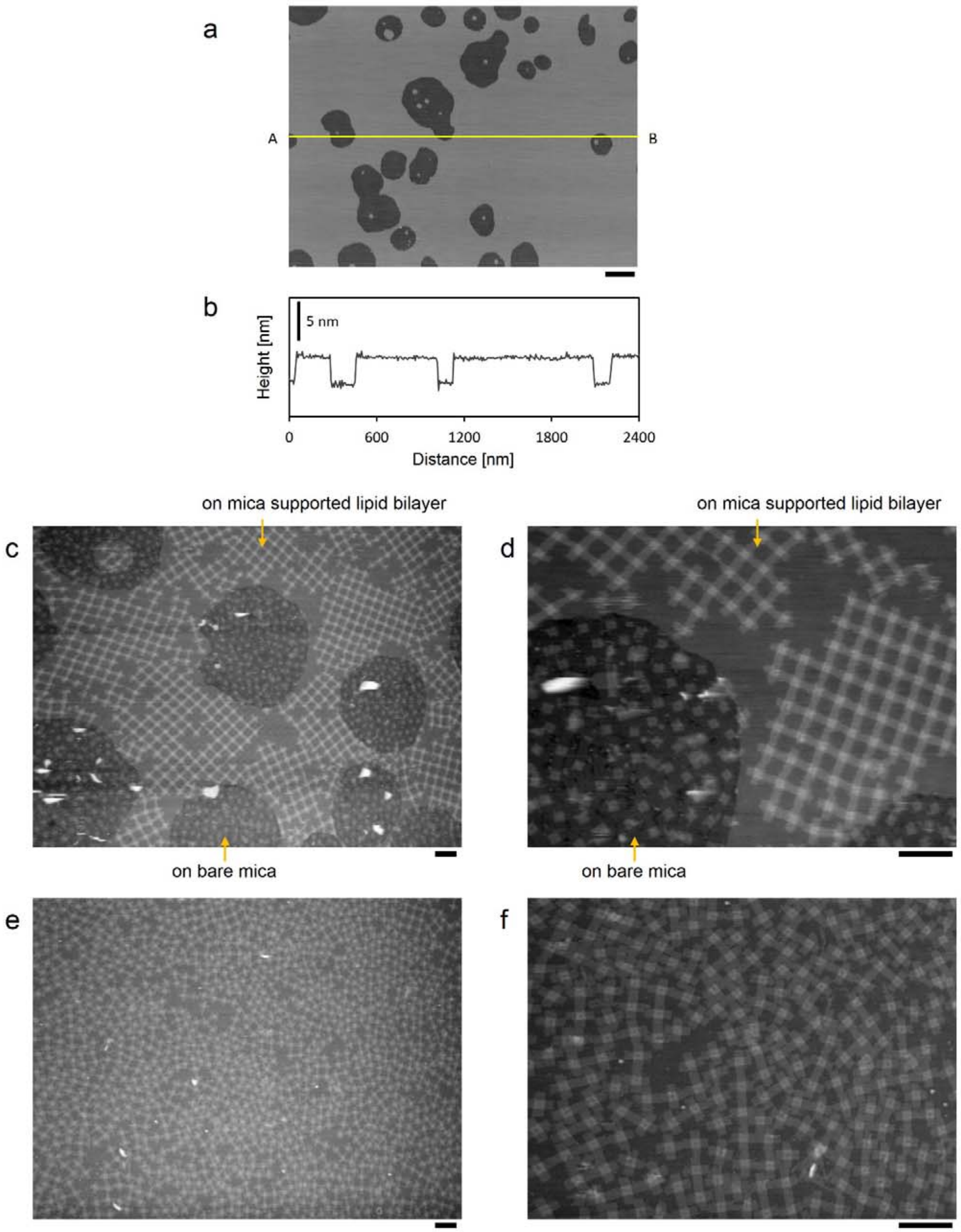
a
A
B
b
5 nm
Height [nm]
0
600
1200
1800
2400
Distance [nm]
on mica supported lipid bilayer
c
on bare mica
on mica supported lipid bilayer
d
on bare mica
e
f

Supplementary Figure 1 | Lattice formation on both SLB and mica surfaces. (**a**) AFM image of the mica supported lipid bilayer (SLB). (**b**) The section profile along the line A–B in (**a**). (**c,d**) AFM images after deposition of the cross-shaped DNA origami units onto the surfaces having both bare mica and SLB regions. Lattices were only observed on the bilayer surface. (**e,f**) AFM image after deposition of the cross-shaped DNA origami units onto the bare mica surface. A drop (2 μL) of cross-shaped DNA origami nanostructures in the standard buffer (20 mM Tris buffer (pH 7.6), 1 mM EDTA, and 10 mM $MgCl_2$) solution (10 nM) was deposited onto the preformed SLB (**c,d**) or freshly cleaved mica (**e,f**) surfaces. After incubation for 60 min at room temperature, the surface was imaged by AFM in ~150 μL of the standard buffer. Note that densities of origamis on bare mica regions in (**c,d**) appeared to be higher than those in (**e,f**). When origami with blunt ends was employed as an assembly unit, the stacking-interaction-mediated formation of the less-densely packed lattices were preferred rather than the close-packing ordering phenomenon. This is probably achieved by kicking out excess origami units into bulk solution or out of bilayer as observed in Fig. 2 and Supplementary Movie 1. The relatively higher density of mica-adsorbed origamis in (**c,d**) may be attributed to the accumulation of these excess units onto the bare mica regions. All scale bars are 200 nm.

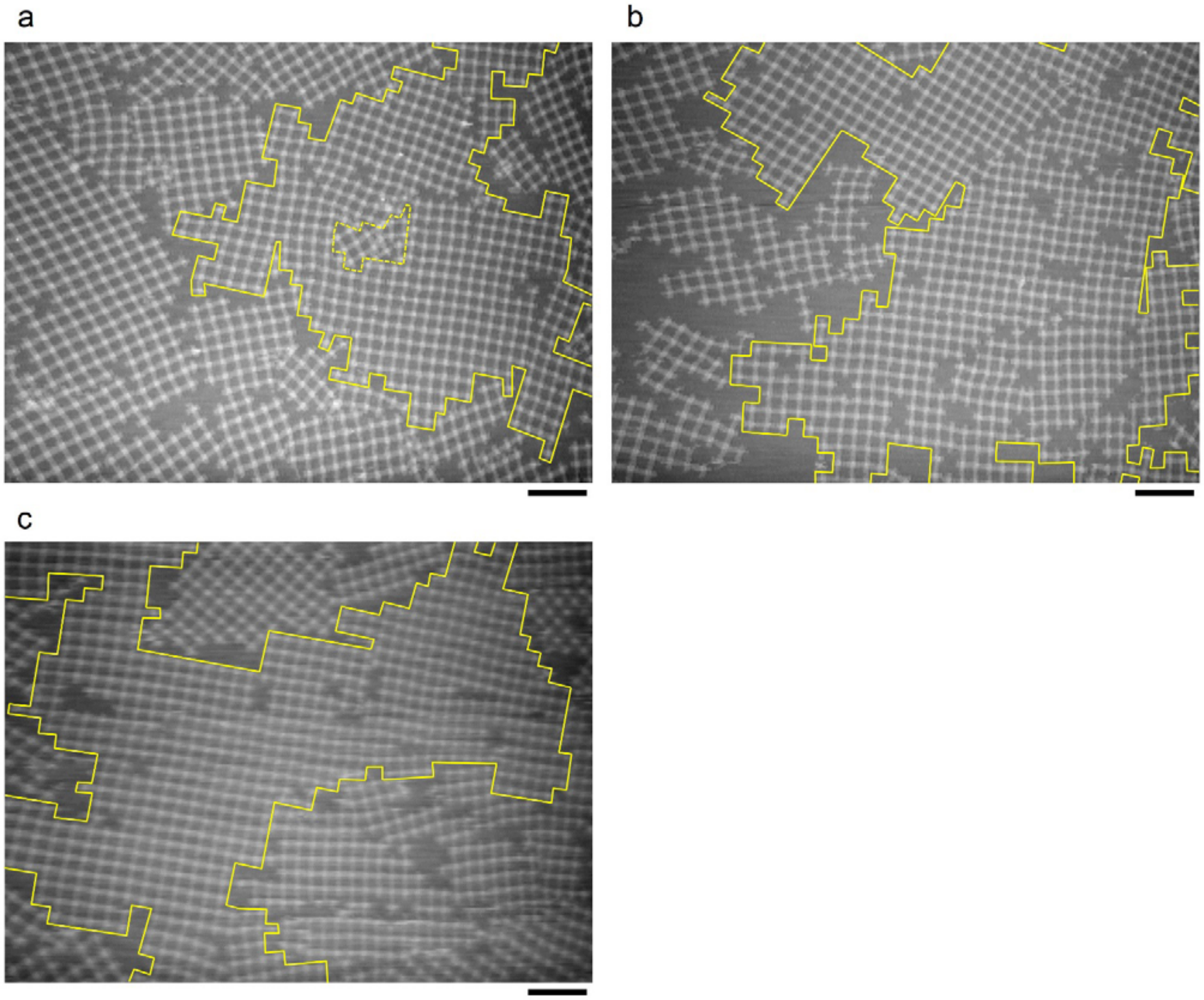

Supplementary Figure 2 │ Large scale AFM images of bilayer-supported lattices made from the cross-shaped DNA origamis with blunt ends. Examples of assemblies having (**a**) ~430, (**b**) ~ 790, and (**c**) ~ 630 origamis in the regions indicated by yellow lines. Origamis surrounded by dashed line in (**a**) was excluded from the counts. Scale bars are 400 nm.

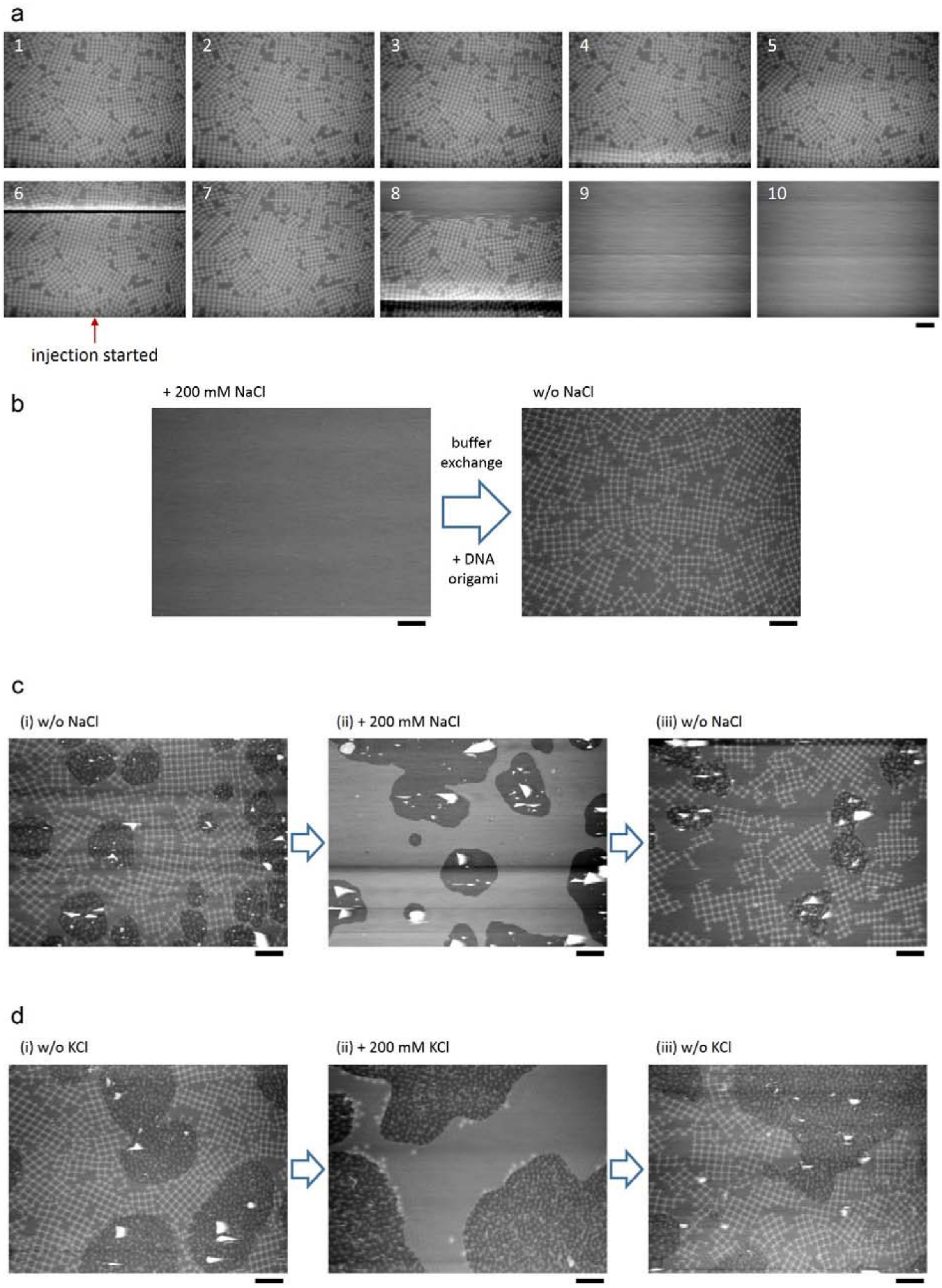
a
1
2
3
4
5
6
7
8
9
10
injection started
b
+ 200 mM NaCl
w/o NaCl
buffer exchange
+ DNA origami
c
(i) w/o NaCl
(ii) + 200 mM NaCl
(iii) w/o NaCl
d
(i) w/o KCl
(ii) + 200 mM KCl
(iii) w/o KCl

Supplementary Figure 3 | Effect of the surface rinsing with NaCl. (**a**) Successive HS-AFM images of salt-induced desorption of lipid bilayer-supported lattices. While scanning of the same area is continued, 15 μL of the buffer containing 2 M NaCl was injected to 135 μL of the standard buffer (20 mM Tris buffer (pH 7.6), 1 mM EDTA, and 10 mM $MgCl_2$), so that the final concentration of NaCl was 200 mM. The lattices were desorbed from the bilayer surface immediately after this treatment. Images were obtained at a scan rate of 0.2 frames per second. (**b**) The reversible effect of NaCl. After the release of cantilever, the buffer containing 200 mM NaCl was gently sucked from the liquid cell of AFM by pipette. Then, a drop (2 μL) of the DNA origami nanostructure in the standard buffer was deposited on to the same surface. After 60 min incubation, the surface was imaged in 135 μL of the standard buffer. (**c**) The reversible effect of NaCl was also tested on the surface having both bare mica and bilayer regions. After the confirmation of the formation of lattices on the bilayer (i), the buffer for AFM imaging was changed to the buffer containing 200 mM NaCl (ii). After the release of cantilever, the buffer containing 200 mM NaCl was gently sucked from the liquid cell of AFM by pipette. Then, a drop (2 μL) of the DNA origami nanostructure in the standard buffer was deposited onto the same surface. After 60 min incubation, the surface was imaged again in 135 μL of the standard buffer (iii). (**d**) The reversible effect of KCl tested on the surface having both bare mica and bilayer regions. The experiment was performed by following the procedure in (**c**) except for the use of KCl instead of NaCl. All scale bars are 400 nm.

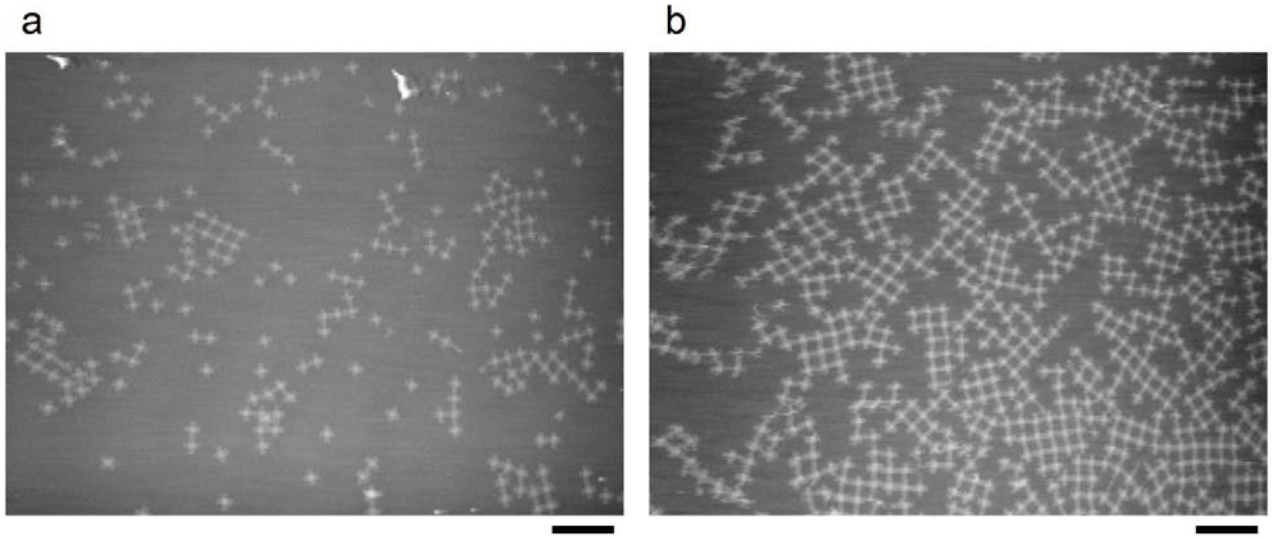

Supplementary Figure 4 │ AFM images of oligomerized cross-shaped origamis. (**a**) AFM image of cross-shaped origamis at lower concentration on the bare mica surface. A drop (2 μL) of diluted cross-shaped DNA origami nanostructures in the standard buffer solution (1 nM) was deposited onto the freshly cleaved mica surface. After incubation for 10 min at room temperature, the surface was imaged by AFM in the standard buffer. Oligomers with 2–12 origamis were observed in addition to monomers. (**b**) AFM image of cross-shaped origamis at standard concentration (10 nM) on the SLB. Image was obtained immediately after the deposition of the origami sample onto the preformed SLB. Oligomers with 3–25 origamis were observed. AFM imaging was performed in the standard buffer. Scale bars are 400 nm.

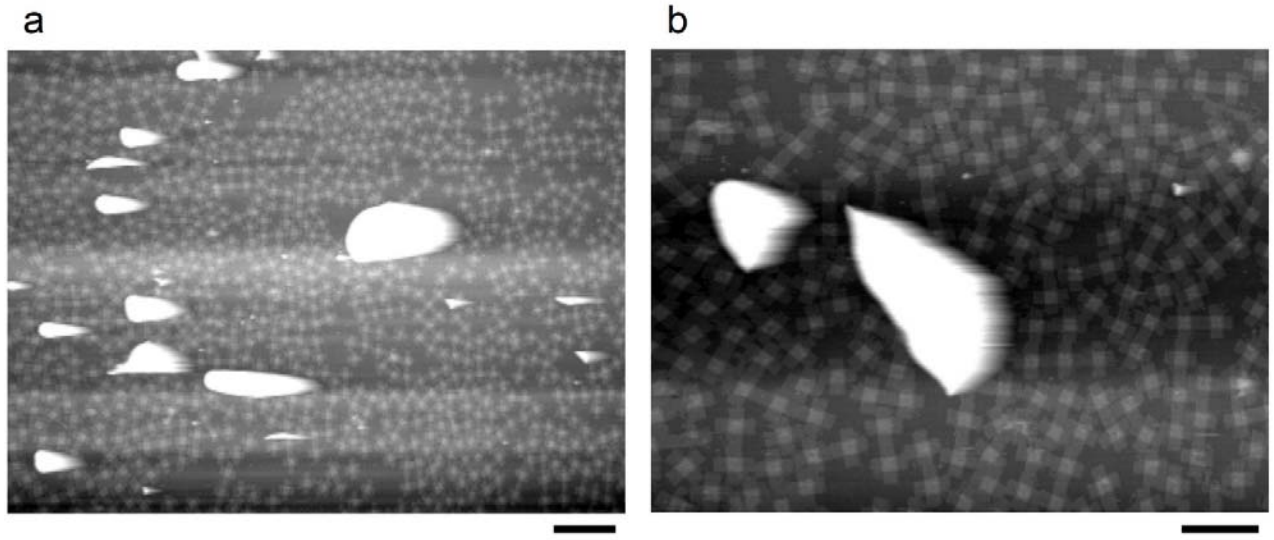

Supplementary Figure 5 │ Large aggregations of cross-shaped origamis with blunt ends. Cross-shaped DNA origami nanostructures in the standard buffer solution (10 nM) was incubated in a test tube for overnight (~12 hours) at room temperature, and then deposited onto the freshly cleaved mica surface. After incubation for 10 min at room temperature, the surface was imaged by AFM in the standard buffer. Scale bars, (**a**) 400 nm and (**b**) 200 nm.

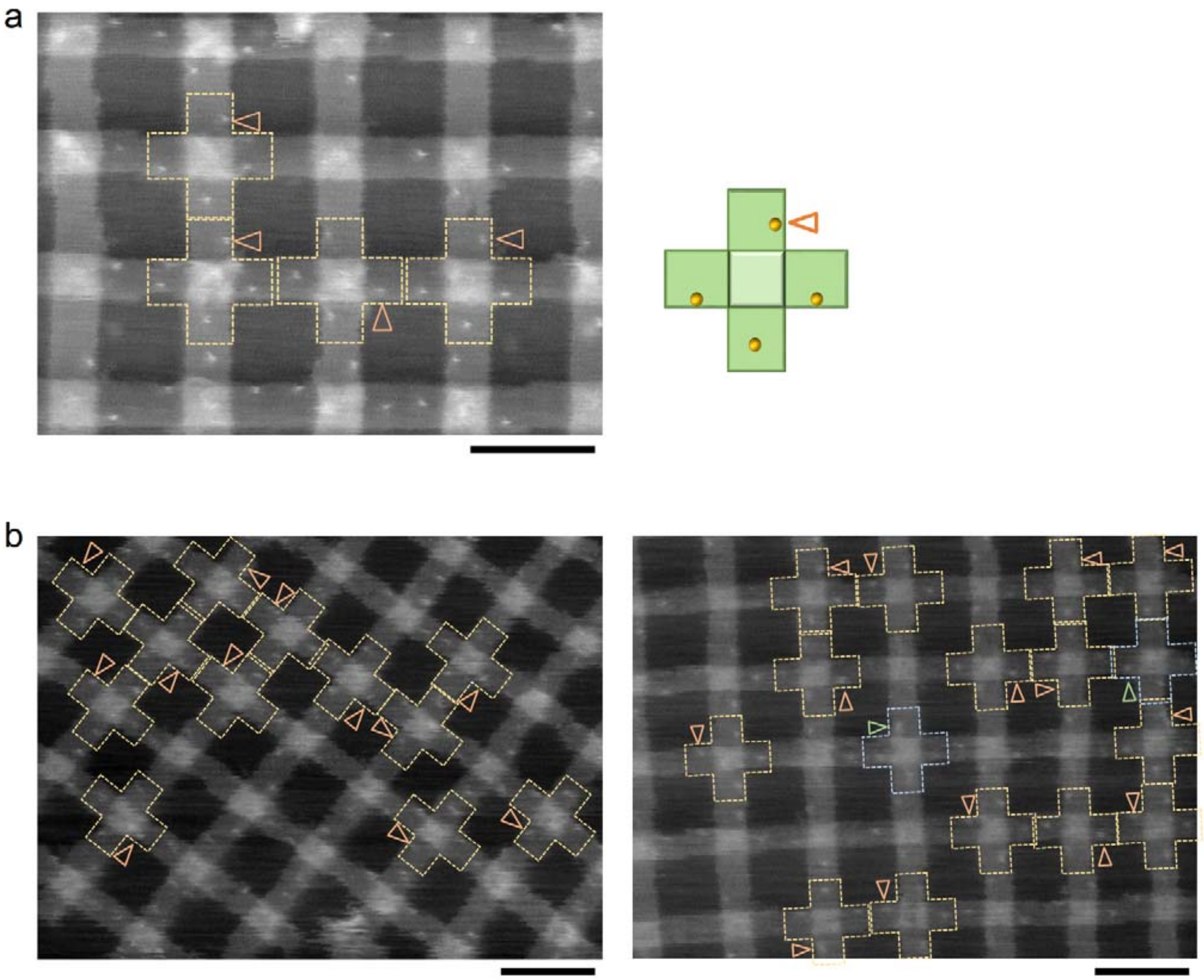

Supplementary Figure 6 │ *In situ* surface modification of the assembled lattice with streptavidin. (**a**) AFM image at 270 s in Fig. 4b. Fully-modified origamis are recognized as facing-up orientation (orange-framed). (**b**) Representative AFM images of the biotinylated lattice after treatment with streptavidin. Origamis in facing-up orientation (orange-framed) and those in facing-down orientation (blue-framed) are indicated. Statistical analysis of AFM images revealed that 47% (116/248) of the origami units had four streptavidin molecules. Among them, 95% (110/116) were recognized as having a 'facing up' orientation. Triangles indicate orientation markers. All scale bars are 100 nm.

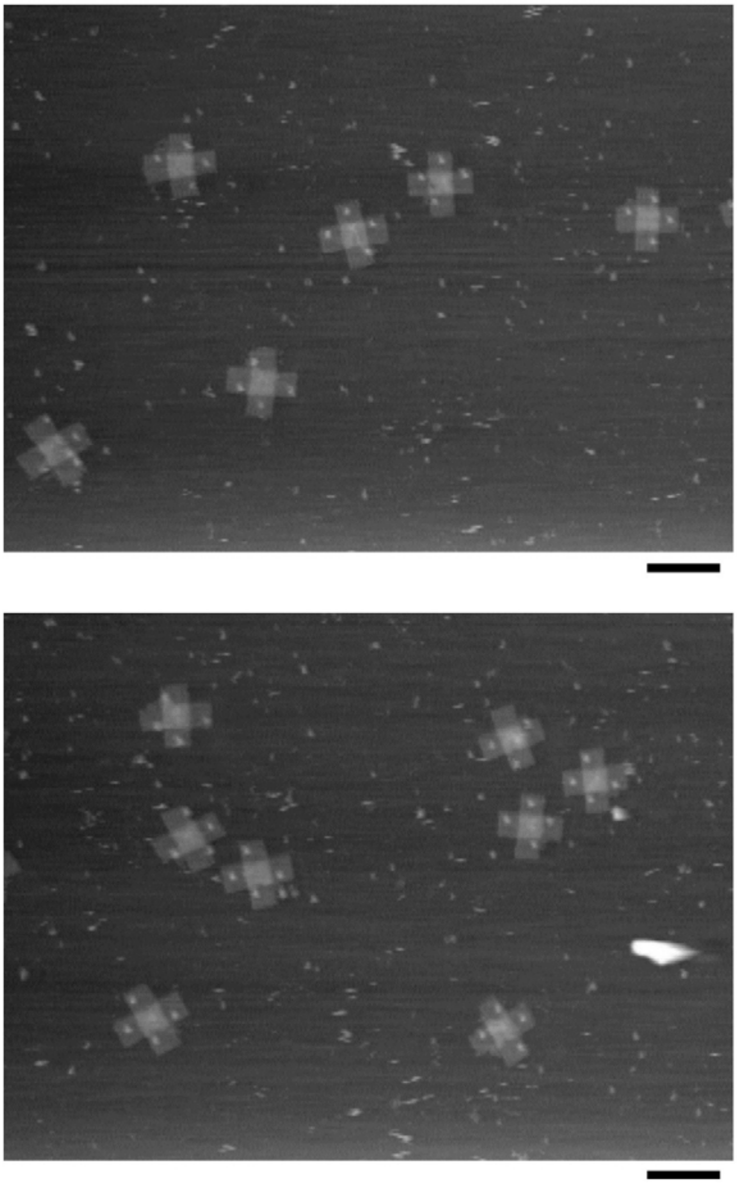

Supplementary Figure 7 │ Surface modification of the cross-shaped origami with streptavidin in test tube. Representative AFM images of streptavidin-decorated cross-shaped DNA origami with T4 tails on bare a mica surface. The binding reaction was performed in 150 μL of solution containing 10 nM DNA origami, 2 μM streptavidin and 20 mM Tris buffer (pH 7.6), 1 mM EDTA, and 10 mM $MgCl_2$ for 5 min incubation at 25 °C. The mixture was deposited on to a bare mica surface and imaged in the standard buffer. High occupancy of the binding sites was achieved with a yield of 92% (435/472). Scale bars are 100 nm.

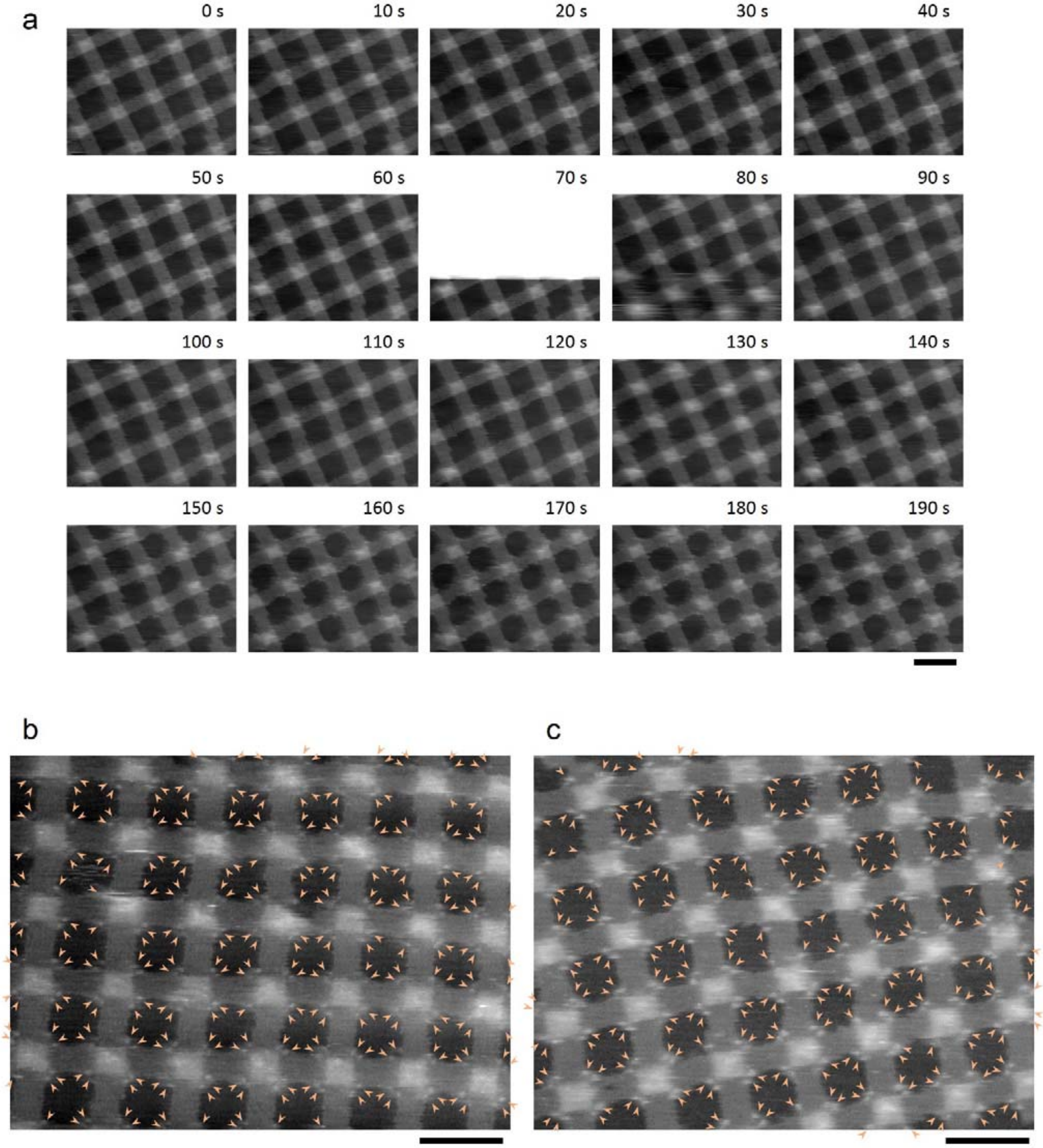
a
0 s
10 s
20 s
30 s
40 s
50 s
60 s
70 s
80 s
90 s
100 s
110 s
120 s
130 s
140 s
150 s
160 s
170 s
180 s
190 s
b
c

Supplementary Figure 8 │ Symmetric displacement of streptavidin molecules. (**a**) Time-lapse AFM images of the modification of the lattice with streptavidin molecules. While scanning of the same area was ongoing, 15 μL of the standard buffer containing 20 μM streptavidin was injected into 135 μL of the imaging buffer (20 mM Tris buffer (pH 7.6), 1 mM EDTA, and 10 mM $MgCl_2$), so that the final concentration of streptavidin was 2 μM. Images were obtained at a scan rate of 0.2 frames per second. The elapsed time is shown in each image. The solution of streptavidin was added at 65 s. Details are seen in Supplementary Movie 4. (**b,c**) Representative AFM images of the biotinylated lattices after the treatment with streptavidin. Yellow arrowheads indicate bound streptavidin molecules. Highly symmetric and specific placement was achieved with a 94% (1185/1256) occupancy of the binding sites. All scale bars are 100 nm.

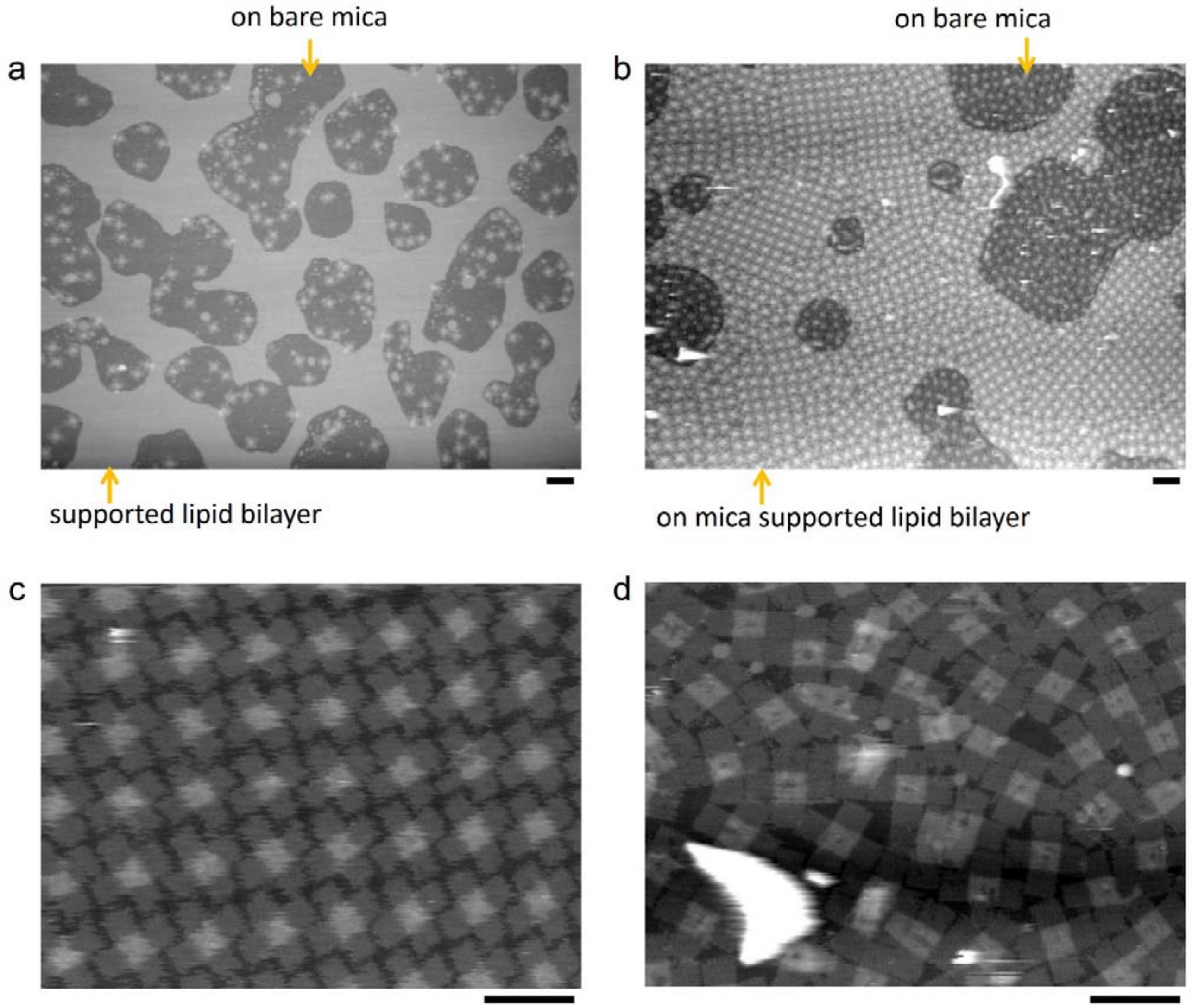

Supplementary Figure 9 │ Different patterns produced by different surface properties. (**a,b**) AFM images after deposition of the stacking-prevented version of cross-shaped DNA origami units onto the surface having both bare mica and SLB regions. A drop (2 μL) of DNA origami nanostructures in the standard buffer solution (**a**: 1 nM, **b**: 10 nM) was deposited onto the surfaces. After incubation for 15 min at room temperature, the surface was imaged by AFM in the standard buffer. (**c**) Zoomed image of the lattice on the SLB region. (**d**) Zoomed image of disordered origamis on the bare mica region. Close-packing was not observed on bare mica surfaces. Scale bars, (**a,b**) 200 nm and (**c,d**) 100 nm.

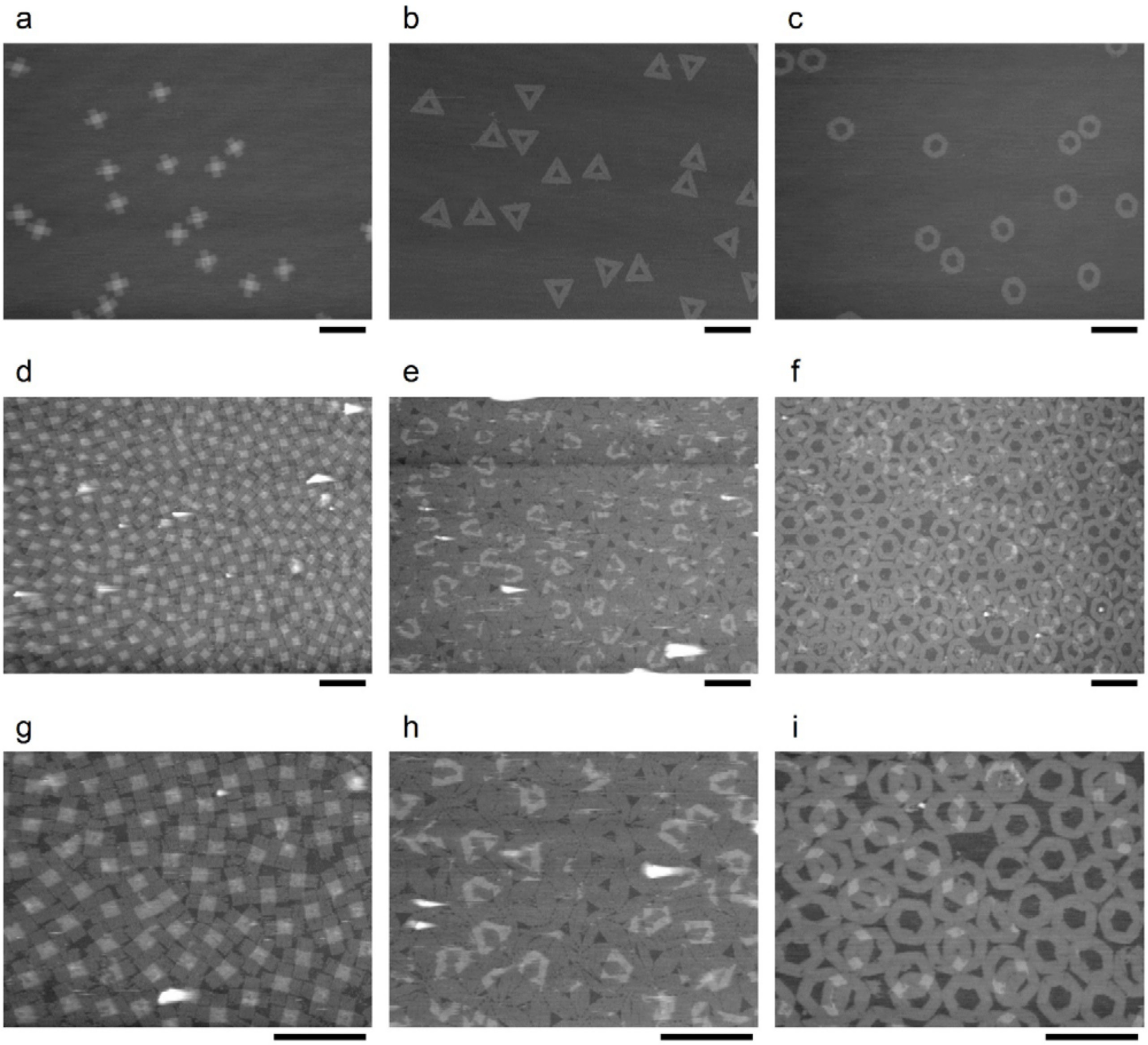

Supplementary Figure 10 │ AFM images of DNA origami nanostructures on bare mica surfaces. A drop (2 μL) of DNA origami nanostructures in the standard buffer solution (**a-c**: 1 nM, **d-i**: 10 nM) was deposited onto the freshly cleaved mica surface. After incubation for 15 min at room temperature, the surface was imaged by AFM in the standard buffer. (**a,d,g**) Cross-shaped DNA origamis, (**b,e,h**) triangular DNA origamis, and (**c,f,i**) hexagonal DNA origamis. All scale bars are 200 nm.

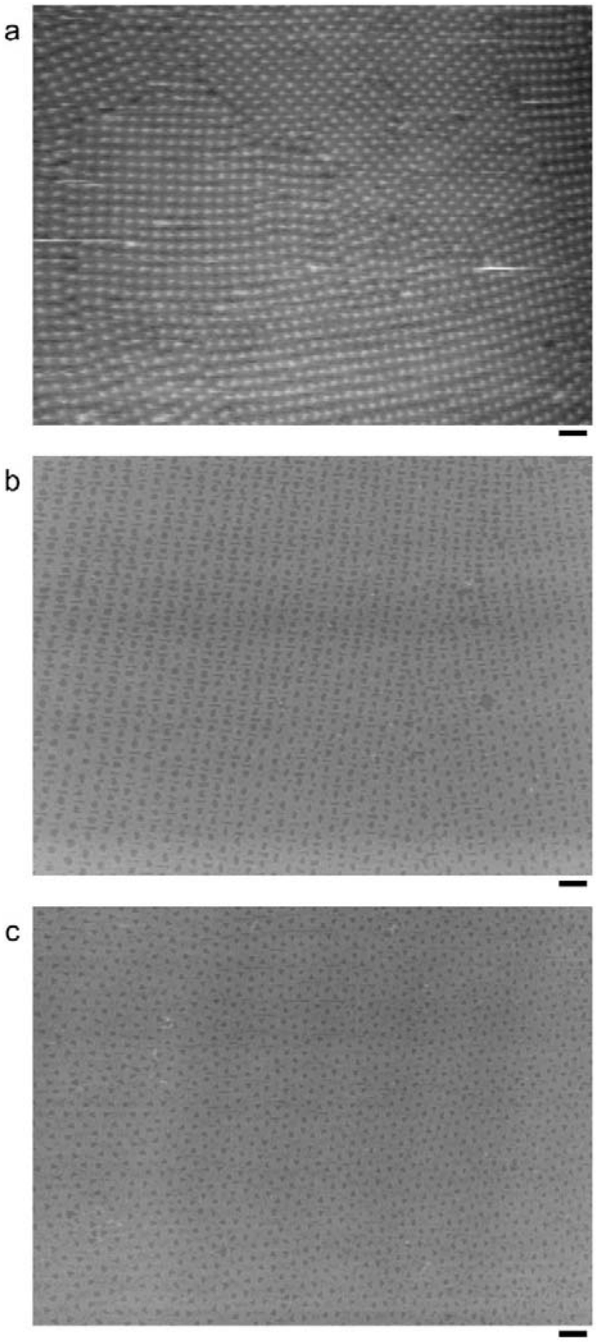

Supplementary Figure 11 │ Large scale AFM images of bilayer-supported close-packed structures. Representative large scale images of close-packed structures of (**a**) cross-shaped origamis with T4 tails, (**b**) triangular origamis, and (**c**) hexagonal origamis. Although domain boundaries of the assemblies are seen in the large scale images, micrometer-sized assemblies are routinely obtained. Scale bars are 200 nm.

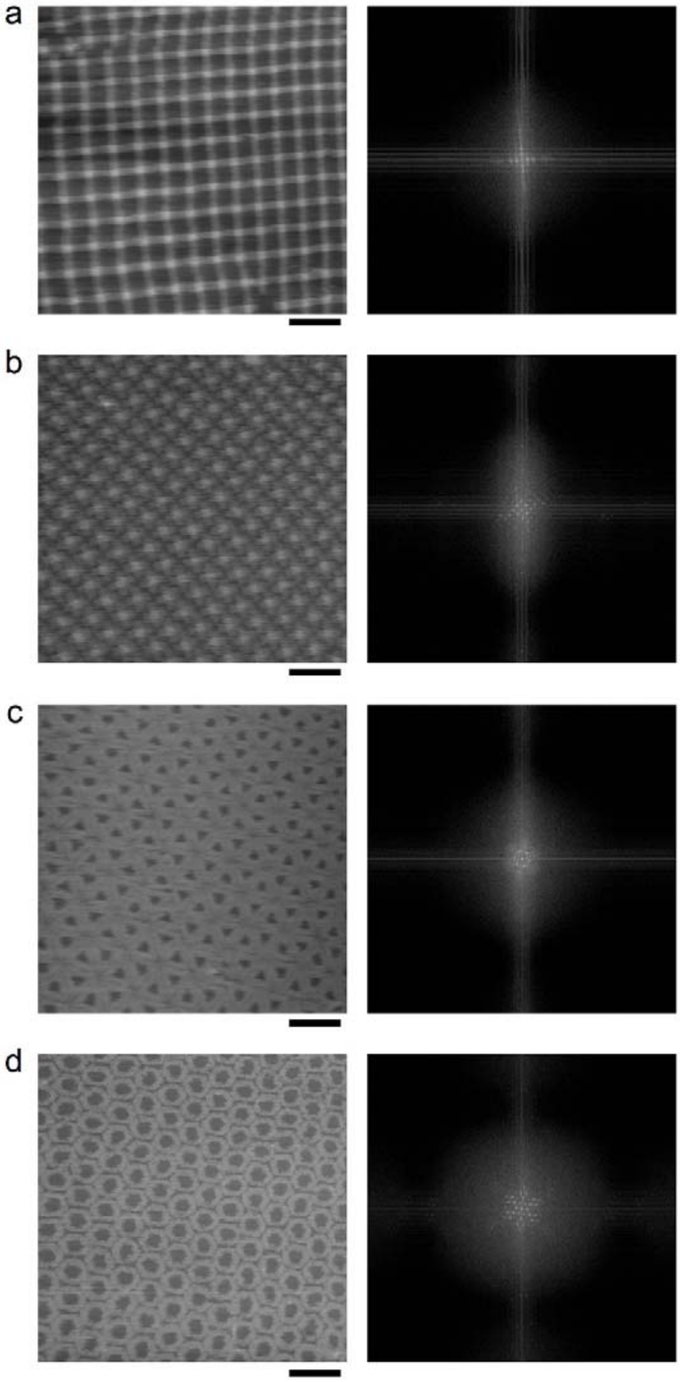

Supplementary Figure 12 │ AFM and FFT images of the DNA origami lattices. AFM and FFT images of the lattices formed by self-assembly of (**a**) cross-shaped origamis with blunt ends, (**b**) cross-shaped origamis with T4 tails, (**c**) triangular origamis, and (**d**) hexagonal origamis. All scale bars are 200 nm.

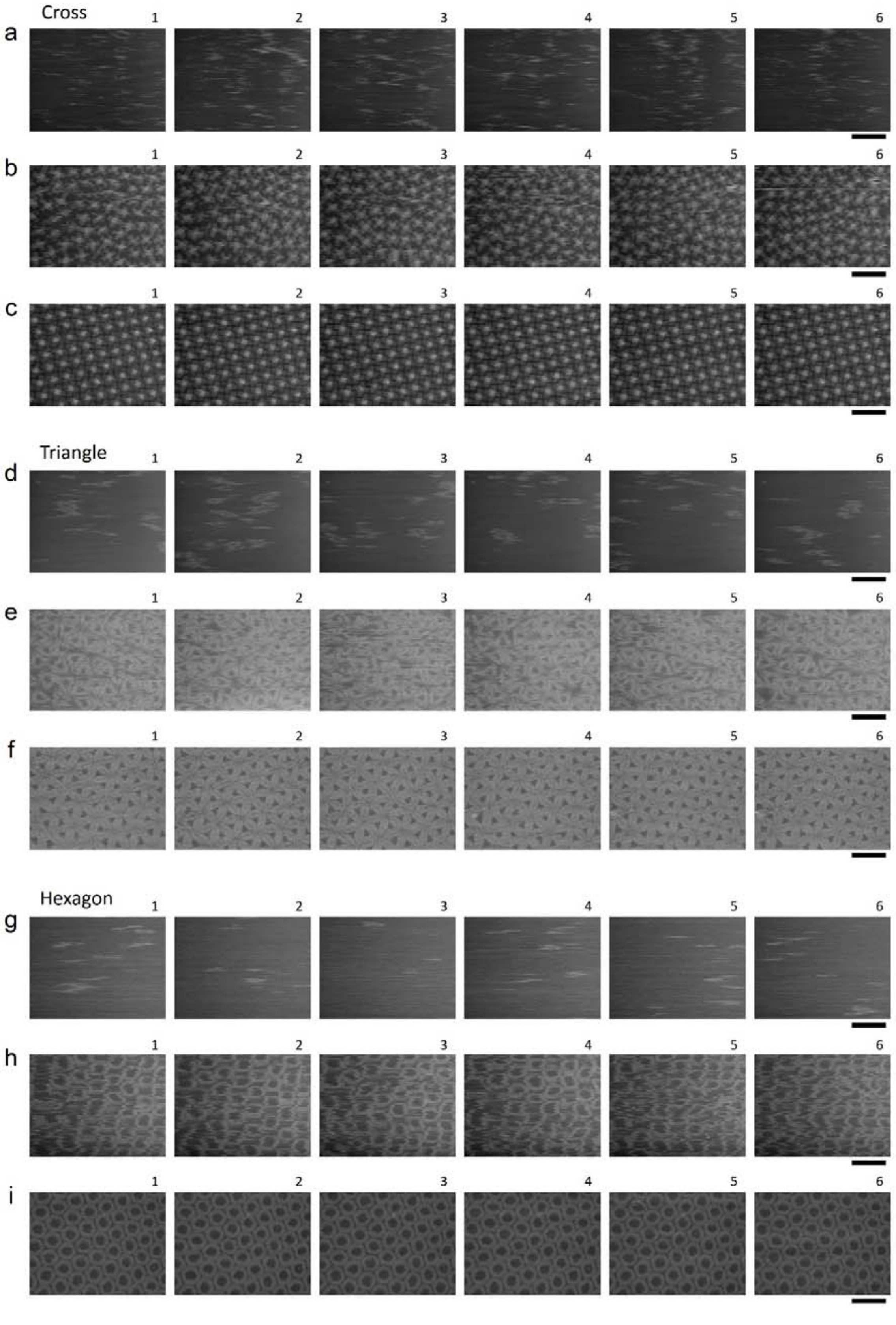
Cross
Triangle
Hexagon
a
b
c
d
e
f
g
h
i
1
2
3
4
5
6

Supplementary Figure 13 │ Treatment of the bilayer surface with various concentration of DNA origami nanostructures. (**a-i**) Time-lapse AFM images after incubation of the following concentrations of DNA origamis: (**a,d,g**) 1 nM, (**b,e,h**) 5 nM, and (**c,f,i**) 10 nM. Samples were prepared in the standard buffer (20 mM Tris buffer (pH 7.6), 1 mM EDTA, and 10 mM $MgCl_2$) as described in Methods section and then imaged in the same buffer. (**a-c**) Cross-shaped DNA origamis with T4 tails. (**d-f**) Triangular DNA origamis. (**g-i**) Hexagonal DNA origamis. Images were obtained at scan rate of (**a,d,g**) 1.0 or (**others**) 0.2 frames per second. Events in (**a**) and (**b**) can be seen in Supplementary Movies 6 and 7. In lower concentrations, origamis diffuse faster than the scan rate, and thus their shapes are not clearly imaged. This smearing effect is attributed to the limited scan rate of the AFM in which the tip is tracking the diffusing origamis. All scale bars are 200 nm.

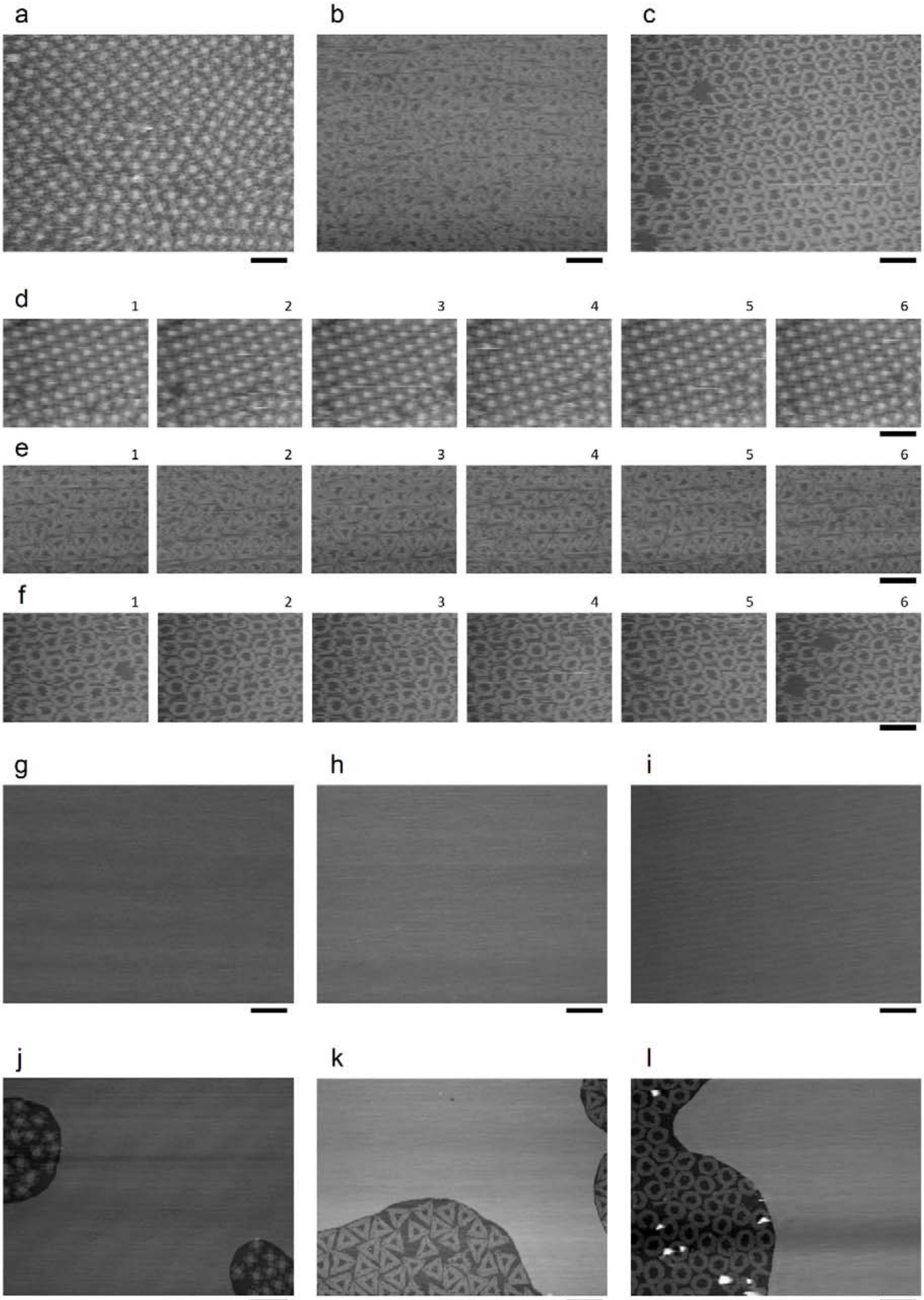
a
b
c
d
1
2
3
4
5
6
e
1
2
3
4
5
6
f
1
2
3
4
5
6
g
h
i
j
k
l

Supplementary Figure 14 │ Effect of Na^+ concentration onto the close packing. Bilayer-supported close-packed structures were first prepared in the standard buffer (20 mM Tris buffer (pH 7.6), 1 mM EDTA, and 10 mM $MgCl_2$) as described in Methods section and then imaged in the buffer containing (**a-f**) 20 mM or (**g-l**) 100 mM NaCl (20 mM Tris buffer (pH 7.6), 1 mM EDTA, 20 mM $MgCl_2$, and 20 mM or 100 mM NaCl). (**a-l**) AFM images of the bilayer surfaces incubated with (**a,d,g,j**) cross-shaped origamis with T4 tails, (**b,e,h,k**) triangular origamis, and (**c,f,i,l**) hexagonal origamis. (**a-c**) AFM images of the bilayer surfaces in the buffer containing 20 mM NaCl. (**d-f**) Time-lapse AFM images of the bilayer surfaces in the presence of 20 mM NaCl. Images were obtained at scan rate of 0.2 frames per second. (**g-i**) AFM images of the bilayer surfaces in the presence of 100 mM NaCl. Origamis were not observed under this condition. (**j-l**) The same experiments with (**g-i**) were performed using the surfaces having both bare mica and SLB regions. Origamis were observed solely on bare mica regions. All scale bars are 200 nm.

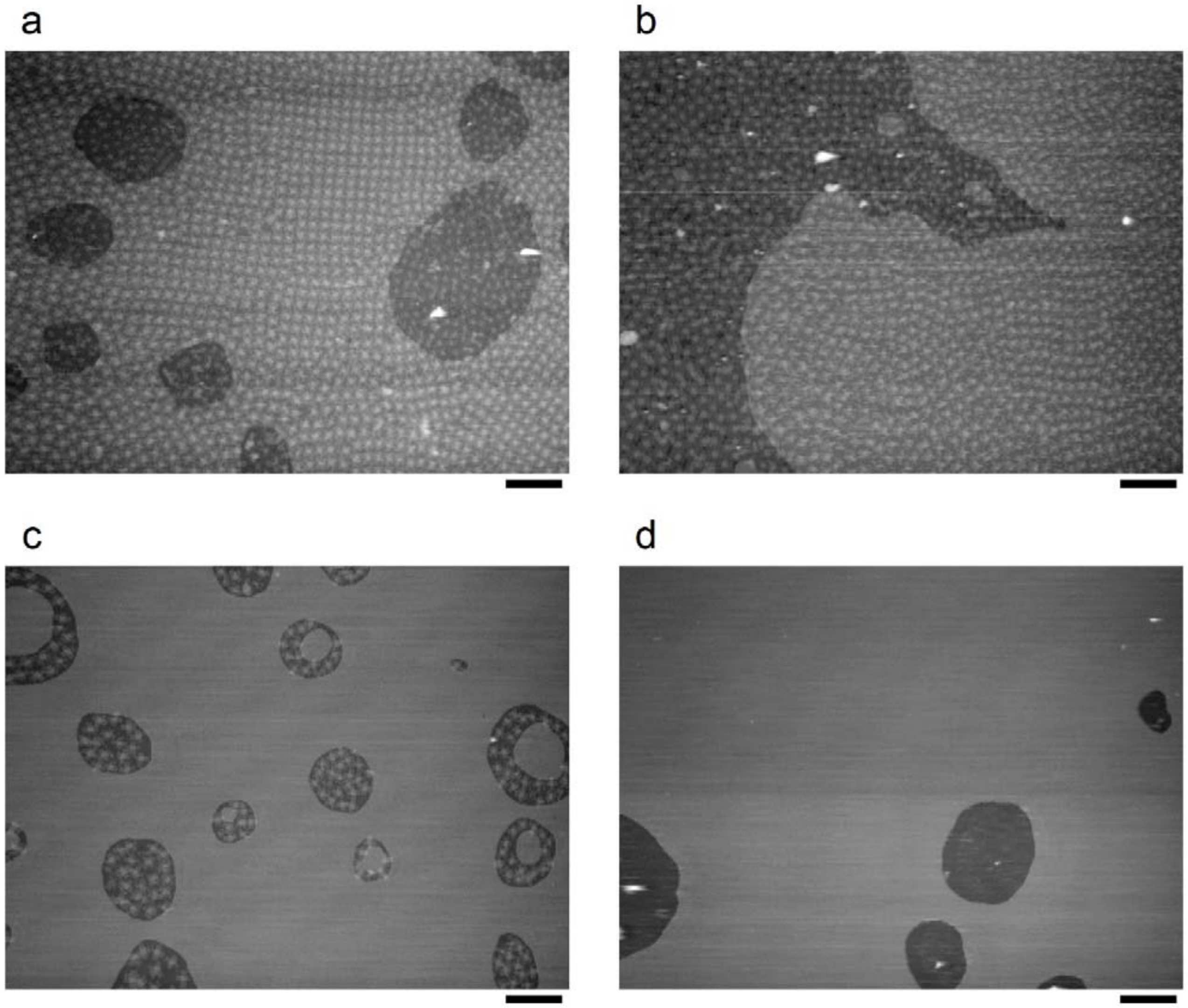

Supplementary Figure 15 │ NaCl induced desorption of origami structures from mica/SLB surfaces. A drop (2 μL) of cross-shaped DNA origamis with T4 tails in the standard buffer solution (10 nM) was deposited onto the surfaces having both bare mica and SLB regions. After incubation for 15 min at room temperature, the surface was imaged by AFM in the buffer containing (**a**) 0 mM, (**b**) 20 mM, (**c**) 100 mM, and (**d**) 200 mM of NaCl. Scale bars are 400 nm.

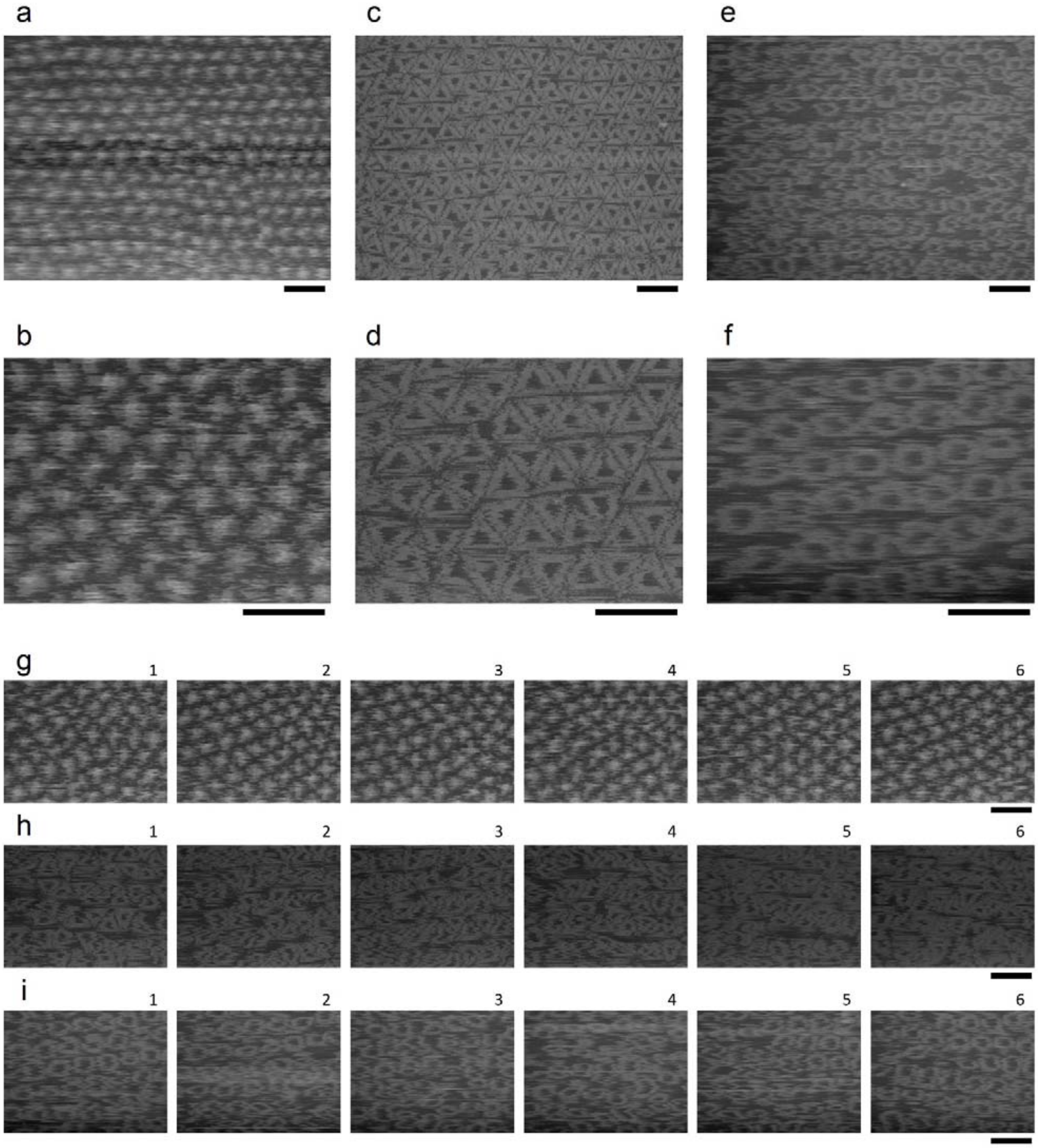
a
c
e
b
d
f
g
h
i
1
2
3
4
5
6

Supplementary Figure 16 | Effect of decrease in Mg^{2+} concentration ([$MgCl_2$]=5 mM) on the close packing. Bilayer-supported close-packed structures were first prepared in the standard buffer (20 mM Tris buffer (pH 7.6), 1 mM EDTA, and 10 mM $MgCl_2$) as described in Methods section and then imaged in low [$MgCl_2$] buffer (20 mM Tris buffer (pH 7.6), 1 mM EDTA, and 5 mM $MgCl_2$). (**a-f**) AFM images of the bilayer surfaces incubated with (**a,b**) cross-shaped origamis with T4 tails, (**c,d**) triangular origamis, and (**e,f**) hexagonal origamis. (**g-i**) Time-lapse AFM images of the bilayer surfaces incubated with (**g**) cross-shaped origamis with T4 tails, (**h**) triangular origamis, and (**i**) hexagonal origamis. Images were obtained at scan rate of 0.5 frames per second. All scale bars are 200 nm.

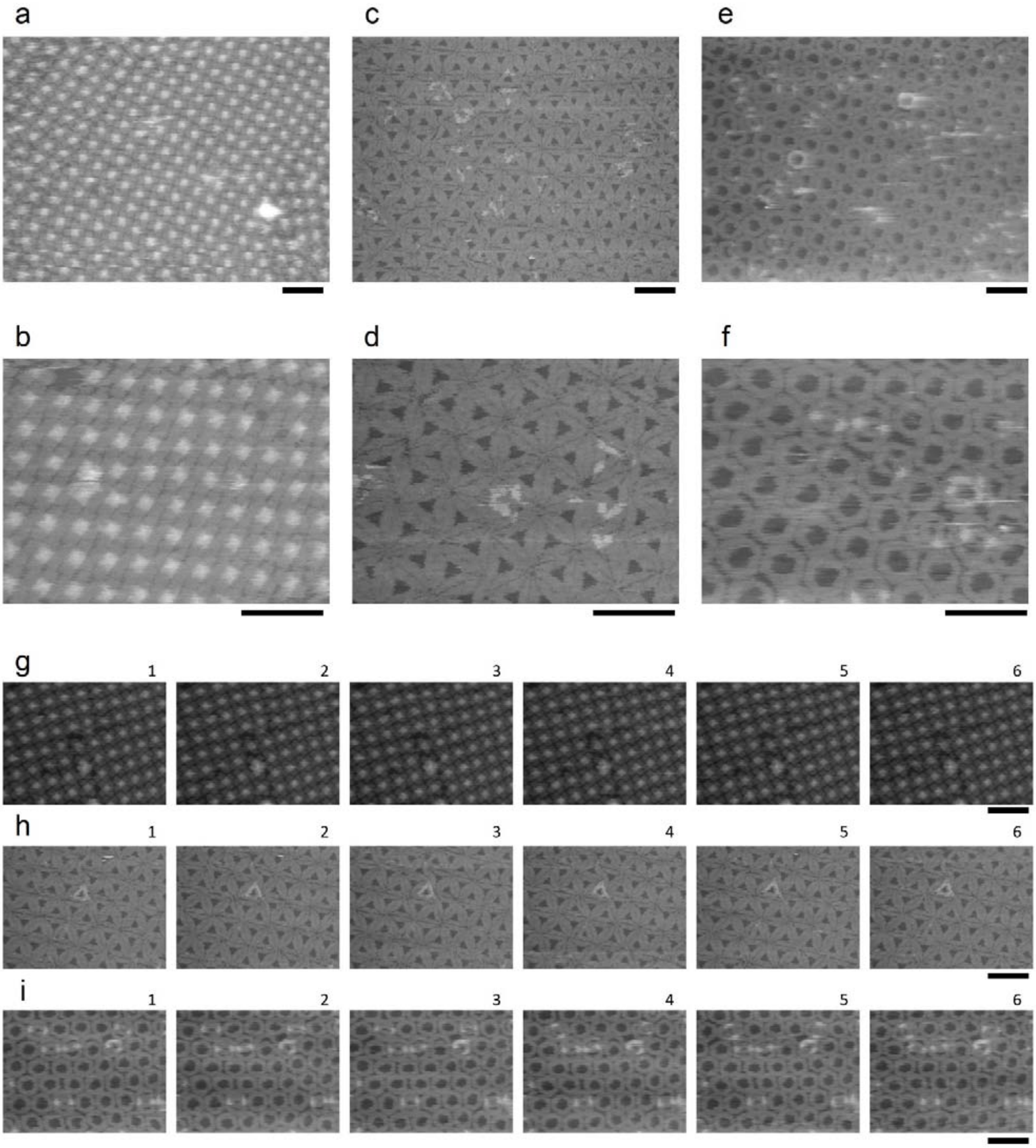
a
c
e
b
d
f
g
1
2
3
4
5
6
h
1
2
3
4
5
6
i
1
2
3
4
5
6

Supplementary Figure 17 | Effect of increase in Mg^{2+} concentration ([$MgCl_2$]=20 mM) onto the close packing. Bilayer-supported close-packed structures were first prepared in the standard buffer (20 mM Tris buffer (pH 7.6), 1 mM EDTA, and 10 mM $MgCl_2$) as described in Methods section and then imaged in high [Mg^{2+}] buffer (20 mM Tris buffer (pH 7.6), 1 mM EDTA, and 20 mM $MgCl_2$). (**a**-**f**) AFM images of the bilayer surfaces incubated with (**a,b**) cross-shaped origamis with T4 tails, (**c,d**) triangular origamis, and (**e, f**) hexagonal origamis. (**g-i**) Time-lapse AFM images of the bilayer surfaces incubated with (**g**) cross-shaped origamis with T4 tails, (**h**) triangular origamis, and (**i**) hexagonal origamis. Images were obtained at scan rate of 0.2 frames per second. All scale bars are 200 nm.

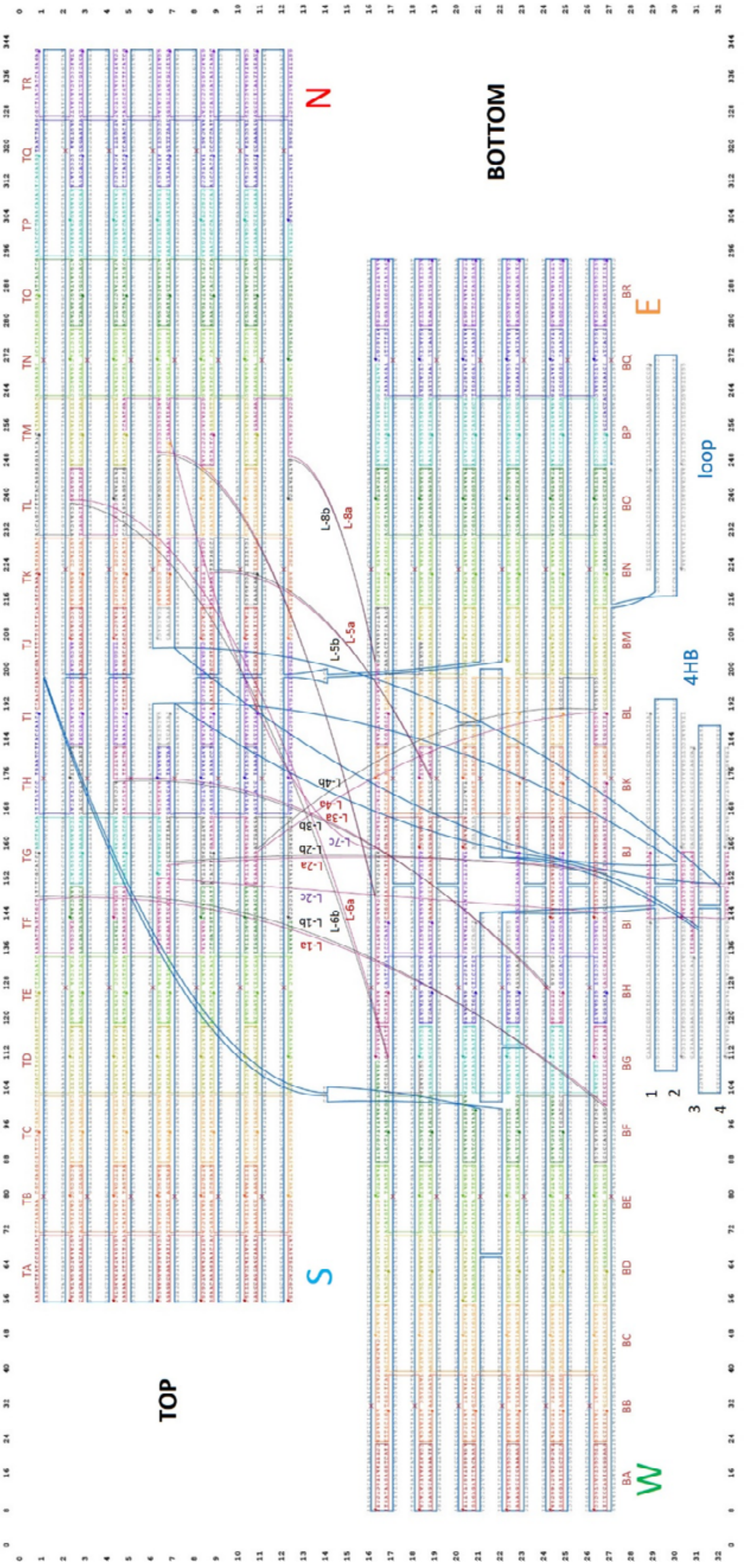

Supplementary Figure 18 | Design of the cross-shaped DNA origami.

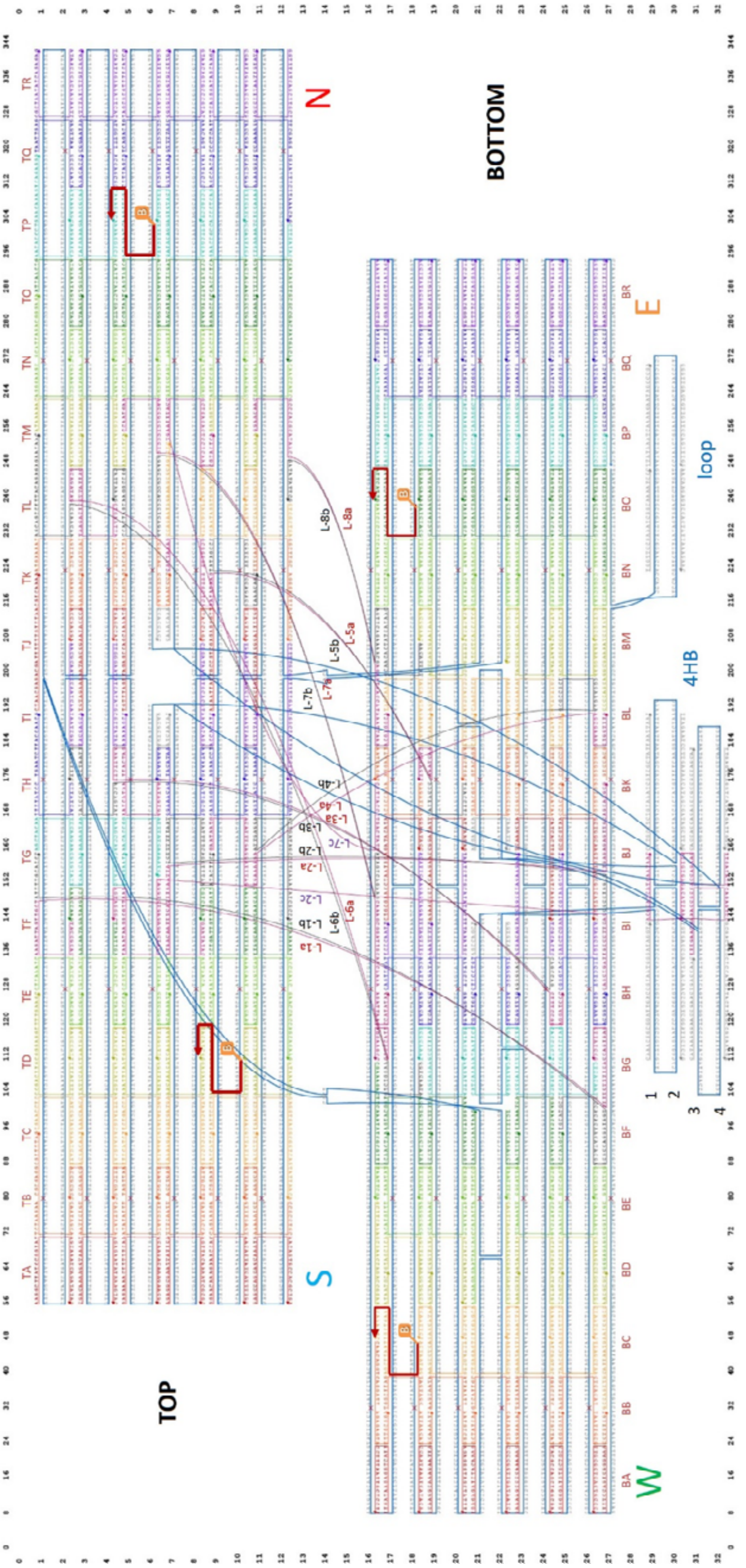

Supplementary Figure 19 | Positions of four biotinylated staple strands for orientation markers.

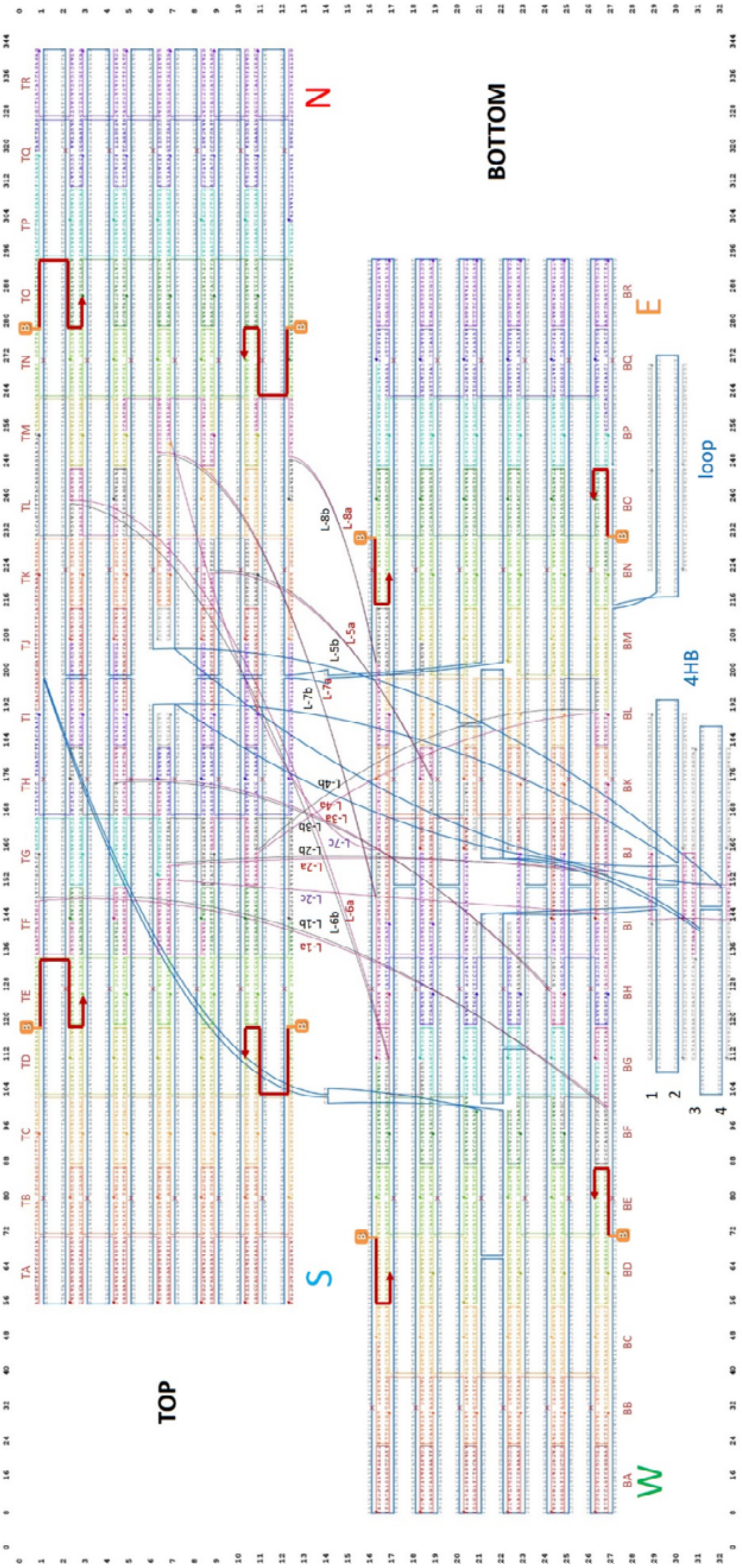

Supplementary Figure 20 | Positions of eight biotinylated staple strands for post-assembly

Supplementary Table 1 | Staple DNA strands for the cross-shaped DNA origami structure.

position	sequence
top-A1	AAGGCTTATCCGGTATAGCAAATCAGATATAG
top-A2	CGACAAAAGGTAAAGTAGAGAATATAAAGTAC
top-A3	GAGAAAACTTTTTCAAGCAAGACAAAGAACGC
top-A4	AGAGGCGAATTATTCAAGTTACAAAATCGCGC
top-A5	GGAACAAAGAAACCACAACATTATCATTTTGC
top-A6	GAGCCAGCAGCAAATGAACAGTGCCACGCTGA
top-B1	CAATAGCATCTAAGAACGCGAGGCGTTTTAG
top-B2	CAGTAATAAATTCTGTCCAGACGACCGCGCC
top-B3	ATCCAATCATATATTTTAGTTAATTTCGAGC
top-B4	GAATACCATTTCAATTACCTGAGTGATGCAA
top-B5	GTTTGAGTCAGAAGGAGCGGAATATTGCTTT
top-B6	CCGCCTGCAAAAATCTAAAGCATTTTTAAAA
top-C1	CGAACCTCCATCGTAGGAATCATTACGACAAT
top-C2	AAACAACAATTTAGGCAGAGGCATTTTCATCT
top-C3	TCTGACCTAACTATATGTAAATGCCAAAAGAA
top-C4	GATGATGAATAACGGATTCGCCTGTATCATCA
top-C5	TATTCCTGGCCCGAACGTTATTAACACCTTGC
top-C6	TGAACCTCGAGGTGAGGCGGTCAGTATTAACA
top-D1	TTTATTTTCCGACTTGCGGGAGGTTTTGAAGC
top-D2	AACATGTATGTTCAGCTAATGCAGAAGCCGTT
top-D3	GGTTATATAAATTTAATGGTTTGAACAACGCC
top-D4	GAGAAACAAACAAACATCAAGAAATTAGGTTG
top-D5	AATCCTTTATTATCAGATGATGGCTACATCGG
top-D6	ATAAAACAAAATATCAAACCCTCATCGTATTA
top-E1	CTTAAATCTCATCGAGAACAAGCAACGCGCC
top-E2	TGTTTATCGAATCGCCATATTTAAATACCGA
top-E3	CCGTGTGATTTTTAACCTCCGGCACAAAATT
top-E4	AATTACATAGTAACAGTACCTTTAATTCATC
top-E5	AATATAATAAACAATTCGACAACATCAATAT
top-E6	CTGGTCAGACCGAACGAACCACCAGCAGAAG
top-F1	TTAATTGAAACAATAGATAAGTCCAAACCAAG
top-F2	GACTTTACCCTGATTGTTTGGATTCGTCAGAT
top-F3	TTAAAAATTTGGCAAATCAACAGTAAGTATTA
top-G1	AGCTACAATCCAAGAACGGGTATTTGAACAAG
top-G2	AAAAATAAAAAGCCAACGCTCAACTAAGAATA
top-G3	AACACCGGATTTATCAAAATCA
top-H1	CTTATCATTTTTATCCTGAATCTTACCAACG
top-H2	AATTGCGTGGAAACAGTACATAAGAAGAGTC
top-H3	TAGATAATGAAGGGTTAGAACCTAATAAAGA
top-H4	ATGCGCGAAGGTTATCTAAAATAGCCGTCAA
top-I1	CTAACGAGACGATTTTTTGTTTAACGTCAAAA
top-I2	TGTAGAAAAATAACGGAATACCCAACCGAGGA
top-I3	TGTTTAGTTAGAGCCAGCAAAATCTGAGCCAT
top-I4	AAAATTATTGCCGTCGAGAGGGTTGTACCAGG
top-I5	AGCACTAACTGTATGGGATTTTGCTTAGTAAA
top-J1	AACGCAATCCAATCAATAATCGGCTACGAGCA
top-J2	TTGGGAATATCATATGCGTTATACAAAAAGCC
top-J3	CGGATAAGTTGCACGTAAAACAGAACCATATC
top-J4	TGAATTTTCAACTAATAGATTAGATCTTTAGG
top-J5	CTTTTGCGTTTTTGAATGGCTATTAGTCTTTA
top-K1	ATGAAAATAGTTACCAGAAGGAAAAAGAACT
top-K2	GGCATGATTCACCGTCACCGACTACCAGTAG
top-K3	CACCATTACCGCCACCCTCAGAGTAAATCCT
top-K4	TTTCAACAGGCTTGCAGGGAGTTAAAGGCCG
top-L1	TAGGATTACCAGAATGGAAAGCGCAGTCTC
top-L2	AAGTTTTGGGAATAGGTGTATCACAGAAGGAT
top-L3	GTCGCTGAGTTTCAGCGGAGTGAGACGATCTA
top-M1	ATAAAAACTTTTTAAGAAAAGTAAGCAGTATG
top-M2	TTAGCAAAATTGACGGAAATTATTAAACGTCA
top-M3	GGAGGTTTCCTCATAGTTAGCGTAAATAGAAA
top-N1	CGAAGCCCAGGGAAGCGCATTAGACGGGAGA
top-N2	AGGTAAATCGTAGAAAATACATATATCTTAC
top-N3	ACCACCGGACCATCGATAGCAGCGGGAGGGA
top-N4	ATTAAGAGGTTCCAGTAAGCGTCCCAGAGCC
top-N5	CAGACAGCAGTACCGCCACCCTCATGAAAGT
top-N6	CAACCATCTAAAGGAATTGCGAAGCATTCCA
top-O1	ATTAACTGATGAAATAGCAATAGCCATAAAGG
top-O2	TGGCAACAACATTCAACCGATTGAACCGTAAT
top-O3	CAGTAGCGATCAAAATCACCGGAAATACATGG
top-O4	CTTTTGATCCTATTATTCTGAAACAGAACCGC
top-O5	CACCCTCAAACTACAACGCCTGTATAATAATT
top-O6	TTTTCACGCGATAGTTGCGCCGACAATGACAA
top-P1	AAGAAACAAACACCCTGAACAAAGTCAGAGGG
top-P2	AAAGGGCGTATAAAAGAAACGCAAATAAGAGC
top-P3	TTTTCATAACAGAATCAAGTTTGCCAAAGACA
top-P4	TTTCGGAAGATACAGGAGTGTACTTTGCCATC
top-P5	CCAGTACAGAACCGCCACCCTCAGCCTGCCTA
top-P6	CTTGATACTTGAAAATCTCCAAAATTTCGTCA
top-Q1	TAATTGAGGAGTTAAGCCCAATAAGACACCA
top-Q2	CGGAATAAATGGTTTACCAGCGCCTTTAGCG

position	sequence
top-Q3	TCAGACTGCCCCCTTATTAGCGTGGTAATAA
top-Q4	GTTTTAACAAACAGTTAATGCCCAGCCACCA
top-Q5	CCCTCATTTACCGTAACACTGAGAAAAGGCT
top-Q6	CCAAAAGGTTCGAGGTGAATTTCTTAAACAG
top-R1	ATAACCCACAAGAATTCGCTAATATCAGAGAG
top-R2	TCAATAGAAAATTCATGTTTATTTTGTCACAA
top-R3	GCATTTTCGGTCATAGTAGCGCGTTTTCATCG
top-R4	GTAACAGTGCCCGTATGGGGTCAGTGCCTTGA
top-R5	CCAATAGGAACCCATGTTCAGGGATAGCAAGC
top-R6	GGTTTATCAGCTTGCTAGCCTTTAATTGTATC
bot-A1	TTGATAAGAGGTCATTCCTTTAATTGCTCCTT
bot-A2	GCAAGGCAAAGAATTACCAATAAATCATACAG
bot-A3	TGAGAGATCTACAAAGAGAGGGTAGCTATTTT
bot-A4	GAAAAGCCCCAAAAACCCCCGGTTGATAATCA
bot-A5	AGGGGGATGTGCTGCATACGCCAGCTGGCGAA
bot-A6	CTTTCCAGTCGGGAAATTGCGCTCACTGCCCG
bot-B1	CAACAGGTCAGGATTAGAGAGTATTTGCGGA
bot-B2	TGGCTTAGTAGTAGCATTAACATGCAAAATT
bot-B3	AAGCAATAATAAATTAATGCCGGGCTATCAG
bot-B4	GTCATTGCTGTCAATCATATGTAAGGAAGAT
bot-B5	TGTATAAGGGGCCTCTTCGCTATAGGCGATT
bot-B6	AAGTTGGGCTCACATTAATTGCGCCTGTCGT
bot-C1	ACTAATAGAGCTTAATTGCTGAATGCAAACTC
bot-C2	TCTAGCTGAAGCCTCAGAGCATAATCAATTCT
bot-C3	AACTAGCACTGAGAGTCTGGAGCATCAACCGT
bot-C4	ATCGGTGCCAAATATTTAAATTGTAATCGTAA
bot-C5	TGAGCTAATAACGCCAGGGTTTTCGAAGGGCG
bot-C6	GCCAGCTGCATTAATGAATCGGCCCTAATGAG
bot-D1	GCTTCAAAGCGAACCAGACCGGAAATAATGCT
bot-D2	GTAGCTCAAGCTGAAAAGGTGGCAAGCTAAAT
bot-D3	CGGTTGTAACCATCAATATGATAT
bot-D4	CAGTTACAAAATACGATGAACGGTAAACGTTA
bot-D5	ATATTTTGGCTGCGCAACTGTTGGCCAGTCAC
bot-D6	GACGTTGTGTAAAGCCTGGGGTGCAACGCGCG
bot-E1	GGGGCGCGACATGTTTTAAATATTAATTCGA
bot-E2	GTCAAATCCCAAAAACATTATGATTTCATTT
bot-E3	AACAAGAGAATAACAGCCATATTATTTATCCCGGAGACA
bot-E4	CCATTCAGTTAAAATTCGCATTATAATTTGC
bot-E5	CATAAAGTAAAACGACGGCCAGTGCCATTCG
bot-E6	GGGAGAGGCGGTTTGCGTATTGGGCCGGAAG
bot-F1	CGAAAGACTTCAAATATCGCGTTTGCAACTAA
bot-F2	AGTACGGTACCTGTTTAGCTATATCCCTGTAA

position	sequence
bot-F3	TACTTTTGAAAGGGTGAGAAAGGCCAATCCAAATAAG
bot-F4	TCTTTCCAGAGCCAATTTTTG
bot-F5	TTAAATCAGGAAACCAGGCAAAGCGCCAAGCT
bot-G1	AAGATTCACGGGAGAAGCCTTTATTCGCAAAT
bot-G2	CAATAGGACATCAAAAATAATTCGTGTAGGTA
bot-G3	CTGGTGCCGCTCATTTTTTATCTGCGTGCAAC
bot-G4	CTCACAATTGCAGGTCGACTCTAGCACCGCTT
bot-H1	CAGTTGATACCATTAGATACATTTTCAACGC
bot-H2	AAGGATAAAATGCCTGAGTAATGCGTCTGGC
bot-H3	CTTCCTGTTGGGCGCATCGTAACCCAGTTTG
bot-H4	CGGGTACCTTCCTGTGTGAAATTACGGGCAA
bot-I1	ACATTCAAGAGTAGATTTAGTTTGTCCCAATT
bot-I2	TAATCTTGCATATATTTTAAATGCAAATTTTT
bot-I3	TCGCACTCACGACAGTATCGGCCTAGAAGAAC
bot-I4	ATAGCTGTGAGCTCGAATTCGTAAGTCACGCT
bot-J1	CTGCGAACCTAATGCAGATACATAGGAATACC
bot-J2	AGAACCCTACAAGAACCGGATATTATCAAGAG
bot-J3	TCAAACTACATCACTTGCCTGAGTCAGGAAGA
bot-J4	GCGCGTAAGCTGGCAAGTGTAGCGTCATGGTC
bot-K1	GAGATTTAACGCCAAAAGGAATTAATCAGGT
bot-K2	GCTGGTATTGTATCATCGCCTGATGAACGGTGTACAGAAGGCTGGC
bot-K3	CCCGCCGCTTTGATTAGTAATAATCGGCCTT
bot-K4	GTCCACGGAGCGGGCGCTAGGGCCCACCACA
bot-L1	TAGTAAGATACAGGTAGAAAGATTCGTAACAA
bot-L2	AGCTGCTCCCCAGCGATTACGCATCCAGGTAC
bot-L3	CAAGCGCGAAAGTACAACGGAGATATATCCAG
bot-L4	AACAATATCGTTGTAGCAATACTTCGCTTAAT
bot-M1	CTTTGACCATTCAGTGAATAAGGCAACGGAAC
bot-M2	ATCGTCACCCTCAACACTCAT
bot-M3	AAATTAACTACCGCCAGCCATTGCGTGGCACAGACAA
bot-M4	GAAAGGAAACAGGGCGCGTACTATCATCACGC
bot-M5	CCCCAGCAGGCGAAAATCCTGTTTCGTGGCGA
bot-N1	AATCCCCCTCAAATGCTTTAAACCCTCGTTT
bot-N2	ACCAGACGTTAATAAAACGAACTTTGCCCTG
bot-N3	ACGAGAAAAAAGAATACACTAAAGCAGCGAA
bot-N4	AGACAGCAAAAGCGTAAGAATACAACAGGAA
bot-N5	AAACGCTCGTAAAAGAGTCTGTCGGTTGCTT
bot-N6	TGACGAGCGGGAAAGCCGGCGAATCCAACGT
bot-O1	AATCTACGACGATAAAAACCAAAAATTCATTG
bot-O2	AAGAGGCACACCAGAACGAGTAGTGGAAGAAA
bot-O3	CTGACCTGTCGGAACGAGGGTAGCTAAAACGA
bot-O4	GCCACCGAATGGAAATACCTACATGAACCCTT

position	sequence
bot-O5	GCTTGACGACGTATAACGTGCTTTTCAGTGAG
bot-O6	CAAAGGGCGAAAAACCGTCTATCAGATTTAGA
bot-P1	CAATACTGCGGAATCGTCATAAATTAGCGAGA
bot-P2	GGCTTTTGACCAGTCAGGACGTTGAAATTGGG
bot-P3	CTTGAGATTACGAAGGCACCAACCAACGGCTA
bot-P4	CAGAGGCTCTGGCCAACAGAGATATTTGACGC
bot-P5	TCAATCGTAGAAGTGTTTTTATAACCTCGTTA
bot-P6	GAATCAGACCTAAAGGGAGCCCCCGGGCGATG
bot-Q1	CTCATTATCAAAAGAAGTTTTGCATAGCGTC
bot-Q2	AATGCCACGGTTTAATTTCAACTAGAACTGG
bot-Q3	GGGACATTTTGAGGACTAAAGACAAATACGT
bot-Q4	GAATCCTGCTGAAATGGATTATTTAATAAAA
bot-Q5	ATCGGAACGCGGGAGCTAAACAGGTACGCCA
bot-Q6	GCCCACTACGTGAACCATCACCCAGCACTAA
bot-R1	AAAATGTTTAGACTGGCAGAGGGGGTAATAGT
bot-R2	ACCTTATGCGATTTTATTAATCATTGTGAATT
bot-R3	TTCCATTAAACGGGTATTTTTCATGAGGAAGT
bot-R4	CCAGTCACACGACCAGTACATTGGCAGATTCA
bot-R5	ATTTTAGACAGGAACGGAGGCCGATTAAAGGG
bot-R6	GGTCGAGGTGCCGTAAAAATCAAGTTTTTTGG
L-1a	TACCGCACAAGATTAGTTGCTtTTTTCTTTTCACCAGTGAGGTTATCCG
L-1b	TGCATGCCTCCACACAACATACGAGCGCCAGGGTGGTtATTTTGCACCC
L-2a	GAATATACTTAACAATTTCATTTGAATTAttCTGAGAGAGTTGCAGCAAGCG
L-2b	CGCCTGGCCtCCTTTTTTAATAGATTTTCAGGTTTAAATACTTCT
L-2c	CAGCTGATTGCCCTTCACttTAGGTCTGAGAGACTACCTAAATAAGGCGTTAAAAGTAGGGC
L-3a	GAATAATGACATTTGAGGATTTAGTGAAAGGAtAGCGAAAGCTGGTTTG
L-3b	GCGCCGCTGGGAAGAAtATTGAGGAACTGATAGCCCTAAAACATCGCCA
L-4a	AATAGTGAAATCATAATTACTAGAAATTCTTtCTTTCCGGAGGATCCC
L-4b	AGGGGACGCAGCCAGtACCAGTATTATCCCATCCTAATTTGTCTTTC
L-5a	CATTAAAGGCGGGGTTTTGCTCAGATATAAGtAAATCAACATCAGTT
L-5b	TGACCTTCCATTACCCtTATAGCCCTCGTCTTTCCAGACGTAAACAAC
L-6a	GTGAATTATAAGACTCCTTATTACGCAGATAGtGTTTCATTAGGAAGCC
L-6b	GGTCAATAGTCTGGAAtCCGAACAAAGCAGCCTTTACAGAGAGAATAAC
L-7a	CCAATGAAAACCGCCTCCCTCtCAAAGCGGATTGCATCAAAAAGATTAAGCCATATAA
L-7b	ATAGTCAGAAGAGAGCCGCCACCCTCtAGAACCATTAGCAAGGCCGGCATTAAAG
L-7c	CTTTACCCTGACTATTttTGAATTTACCGCTGAGACTCCTCAAGCGTACTCA
L-8a	GGAACAACGCCCACGCATAACCtAGAATGACCATAAATCAAAACGAGGCA
L-8b	AACATTATGCAACACTATCATAACAGTTCAGAAAACGtGATATATTCG
4HB1	GGATTGACCGTAATGGAGTAACAATGACCAAC
4HB2	CTTGCTTCTAGCTTAGCACCACCA
4HB3	TGTGAGCGGATAGGTCGCGACCTGCTCCATGT
4HB4	TTAGAATCCTTGAAAACATAGCGATGTAAATCGGTCAGACGATTGGCC
4HB5	CGGTCAATCATAAGGGCGGAACGACAAACGGC
4HB6	TTTGAACGAAACCGCCCAGAGTCCACGCATCATGAATAAC
4HB7	GAGCCGCCGCCAGCATTTGAGGCAGTCGCTATTAATTAAT
4HB8	TACTTAGCAACCGAACCCCGTCGGATTCTCCG
4HB9	TTGATATTACCAGAACATTGTGAGACATTAAA
loop1	TGGTTCCGAAATCGGCAAAATCCC
loop2	TTATAAATCAAAAGAATAGCCCGA
loop3	AACAAGAGTCCACTATTAAAGAAC
loop4	GGGTTGAGTGTTGTTCCAGTTTGG

Supplementary Table 2 │ Staple DNA strands for preventing stacking interaction.

position	sequence
top-A1-L	ttttAAGGCTTATCCGGTATAGCAAATCAGATATAGtttt
top-A2-L	ttttCGACAAAAGGTAAAGTAGAGAATATAAAGTACtttt
top-A3-L	ttttGAGAAAACTTTTTCAAGCAAGACAAAGAACGCtttt
top-A4-L	ttttAGAGGCGAATTATTCAAGTTACAAAATCGCGCtttt
top-A5-L	ttttGGAACAAAGAAACCACAACATTATCATTTTGCtttt
top-A6-L	ttttGAGCCAGCAGCAAATGAACAGTGCCACGCTGAtttt
top-R1-L	ttttATAACCCACAAGAATTCGCTAATATCAGAGAGtttt
top-R2-L	ttttTCAATAGAAAATTCATGTTTATTTTGTCACAAtttt
top-R3-L	ttttGCATTTTCGGTCATAGTAGCGCGTTTTCATCGtttt
top-R4-L	ttttGTAACAGTGCCCGTATGGGGTCAGTGCCTTGAtttt
top-R5-L	ttttCCAATAGGAACCCATGTTCAGGGATAGCAAGCtttt
top-R6-L	ttttGGTTTATCAGCTTGCTAGCCTTTAATTGTATCtttt
bot-A1-L	ttttTTGATAAGAGGTCATTCCTTTAATTGCTCCTTtttt
bot-A2-L	ttttGCAAGGCAAAGAATTACCAATAAATCATACAGtttt
bot-A3-L	ttttTGAGAGATCTACAAAGAGAGGGTAGCTATTTTtttt
bot-A4-L	ttttGAAAAGCCCCAAAAACCCCCGGTTGATAATCAtttt
bot-A5-L	ttttAGGGGGATGTGCTGCATACGCCAGCTGGCGAAtttt
bot-A6-L	ttttCTTTCCAGTCGGGAAATTGCGCTCACTGCCCGtttt
bot-R1-L	ttttAAAATGTTTAGACTGGCAGAGGGGGTAATAGTtttt
bot-R2-L	ttttACCTTATGCGATTTTATTAATCATTGTGAATTtttt
bot-R3-L	ttttTTCCATTAAACGGGTATTTTTCATGAGGAAGTtttt
bot-R4-L	ttttCCAGTCACACGACCAGTACATTGGCAGATTCAtttt
bot-R5-L	ttttATTTTAGACAGGAACGGAGGCCGATTAAAGGGtttt
bot-R6-L	ttttGGTCGAGGTGCCGTAAAAATCAAGTTTTTTGGtttt

Supplementary Table 3 | Staple DNA strands with biotin for streptavidin labeling.

position	Sequence (X=biotin)
top-D5 biotin	XttAATCCTTTATTATCAGATGATGGCTACATCGG
top-P3 biotin	XttTTTTCATAACAGAATCAAGTTTGCCAAAGACA
bot-C1 biotin	XttACTAATAGAGCTTAATTGCTGAATGCAAACTC
bot-O1 biotin	XttAATCTACGACGATAAAAACCAAAAATTCATTG

Supplementary Table 4 | Staple DNA strands with biotin for periodic positioning of streptavidin.

position	Sequence (X=biotin)
top-D1'	TTTATTTTCCGACTTGCGGGAGGT
top-E1 biotin	XTTTGAAGCCTTAAATCTCATCGAGAACAAGCAACGCGCC
top-D6 biotin	XAGCAGAAGATAAAACAAAATATCAAACCCTCATCGTATTA
top-E6'	CTGGTCAGACCGAACGAACCACC
top-N1'	CGAAGCCCAGGGAAGCGCATTAG
top-O1 biotin	XACGGGAGAATTAACTGATGAAATAGCAATAGCCATAAAGG
top-N6 biotin	XAATGACAACAACCATCTAAAGGAATTGCGAAGCATTCCA
top-O6'	TTTTCACGCGATAGTTGCGCCGAC
bot-D1 biotin	XGCGAACCAGACCGGAAATAATGCT
bot-E1'	GGGGCGCGACATGTTTTAAATATTAATTCGAGCTTCAAA
bot-D6'	GACGTTGTGTAAAGCCTGGGGTGCAACGCGCGGGGAGAGG
bot-E6 biotin	XCGGTTTGCGTATTGGGCCGGAAG
bot-N1 biotin	XTCAAATGCTTTAAACCCTCGTTT
bot-O1'	AATCTACGACGATAAAAACCAAAAATTCATTGAATCCCCC
bot-N6'	TGACGAGCGGGAAAGCCGGCGAATCCAACGTCAAAGGGC
bot-O6 biotin	XGAAAAACCGTCTATCAGATTTAGA
